# A highly secreted sulphamidase engineered to cross the blood-brain barrier corrects brain lesions of mice with mucopolysaccharidoses type IIIA

**DOI:** 10.1002/emmm.201202083

**Published:** 2013-04-09

**Authors:** Nicolina Cristina Sorrentino, Luca D'Orsi, Irene Sambri, Edoardo Nusco, Ciro Monaco, Carmine Spampanato, Elena Polishchuk, Paola Saccone, Elvira De Leonibus, Andrea Ballabio, Alessandro Fraldi

**Affiliations:** 1 Telethon Institute of Genetics and Medicine (TIGEM)Naples, Italy; 2 Institute of Genetics and Biophysics (IGB)Naples, Italy; 3 Medical Genetics, Department of Pediatrics, Federico II UniversityNaples, Italy; 4 Department of Molecular and Human Genetics, Baylor College of MedicineHouston, TX, USA; 5 Jan and Dan Duncan Neurological Research Institute, Texas Children's HospitalHouston, TX, USA

**Keywords:** blood-brain barrier, CNS therapy, lysosomal storage disorders, MPS-IIIA, sulphamidase

## Abstract

Mucopolysaccharidoses type IIIA (MPS-IIIA) is a neurodegenerative lysosomal storage disorder (LSD) caused by inherited defects of the sulphamidase gene. Here, we used a systemic gene transfer approach to demonstrate the therapeutic efficacy of a chimeric sulphamidase, which was engineered by adding the signal peptide (sp) from the highly secreted iduronate-2-sulphatase (IDS) and the blood-brain barrier (BBB)-binding domain (BD) from the Apolipoprotein B (ApoB-BD). A single intravascular administration of AAV2/8 carrying the modified sulphamidase was performed in adult MPS-IIIA mice in order to target the liver and convert it to a factory organ for sustained systemic release of the modified sulphamidase. We showed that while the IDS sp replacement results in increased enzyme secretion, the addition of the ApoB-BD allows efficient BBB transcytosis and restoration of sulphamidase activity in the brain of treated mice. This, in turn, resulted in an overall improvement of brain pathology and recovery of a normal behavioural phenotype. Our results provide a novel feasible strategy to develop minimally invasive therapies for the treatment of brain pathology in MPS-IIIA and other neurodegenerative LSDs.

## INTRODUCTION

Mucopolysaccharidosis type IIIA (MPS-IIIA or Sanfilippo disease) is a neurodegenerative disorder caused by deficiency of the lysosomal enzyme sulphamidase (SGSH), which is responsible for catabolizing heparan sulphate (HS). MPS-IIIA is characterized by progressive neurodegeneration with subsequent mental decline and a significantly shortened lifespan (often <20 years). Clinical symptoms include hyperactivity, aggressive behaviour, and sleep disturbance (Hemsley & Hopwood, 2005). A naturally occurring mouse model of MPS-IIIA, which presents a phenotype resembling the human form of the disease has been identified and characterized (Bhattacharyya et al, 2001; Bhaumik et al, 1999; Fraldi et al, 2007).

The treatment of brain pathology is the main goal and hurdle for the development of a therapeutic approach for MPS-IIIA. The most direct way to access the brain tissue is through direct intraventricular/intracerebral injections of the therapeutic molecule. We have successfully used this approach by developing and testing a gene therapy protocol in newborn MPS-IIIA mice based on the intracerebral injection of an AAV vector encoding for both *SGSH* and *SUMF1* gene (Fraldi et al, 2007). In addition, enzyme replacement therapy (ERT) protocols have been tested based on the administration of a recombinant sulphamidase enzyme through direct brain injection in MPS-IIIA mice (Hemsley et al, 2007, 2008; Savas et al, 2004). Although these direct brain-targeting approaches have been shown to be therapeutically relevant, they represent highly invasive approaches and thus have limited appeal for future human therapeutic applications.

Since every neuron in the brain is perfused by its own blood vessel, an effective minimally invasive alternative route to target the brain could be the intravenous administration of the therapeutic molecule (Pardridge, 2002a). However, the very dense microvasculature network that forms the blood-brain barrier (BBB) is not permeable to all molecules and might impede the effective delivery of therapeutic agents to brain tissue (Pardridge, 2005). In fact, although the intravenous administration of a lysosomal enzyme has resulted in the therapeutic benefit on the somatic pathology of many lysosomal storage disorders (LSDs), it has had limited or no effect on central nervous system (CNS) pathology because the BBB has proven to be impermeable for large molecules (Brady & Schiffmann, 2004). Interestingly, some studies have indicated that overloading the blood stream with a lysosomal enzyme might lead to a partial passive cross of the BBB (Polito & Cosma, 2009; Vogler et al, 2005); however, the resulting effect on brain pathology is modest and the mechanisms underlying the transport across the BBB remain unclear (Urayama et al, 2004). Intravenous injection of sulphamidase in MPS-IIIA mice does not improve brain pathology or behavioural abnormalities when the enzyme is supplied subsequent to the first 2 weeks of age after the BBB has became impermeable (Gliddon & Hopwood, 2004). These strategies are only effective when administered to younger mice with a permeable BBB (Gliddon & Hopwood, 2004). Consistently, sulphamidase has been shown to be transported across the BBB in neonatal mice through the mannose 6-phosphate (M6P) receptor-mediated pathway, however the influx of sulphamidase into the adult brain was negligible due to the reduced expression of M6P receptors in the cells forming the BBB (Urayama et al, 2008). Therefore, in this scenario the real challenge to establishing a therapeutic treatment for MPS-IIIA, and for other LSDs that involve the CNS, is developing systemic treatment strategies that can overcome the obstacle posed by the BBB. An effective strategy for crossing the BBB is to target proteins in the CNS via transcytosis, a process mediated by receptors that are highly and age-independently enriched on the BBB such as the low-density lipoprotein receptor (LDLR) and the transferrin receptor (TfR; Pardridge, 2002b). Recently, TfR-mediated transcytosis of therapeutic antibodies has been demonstrated to be potentially effective in treating Alzheimer disease (Atwal et al, 2011; Yu et al, 2011). Notably, these approaches were based on the reduction of the antibody affinity for the transferrin receptors that facilitated the trancytosis thus increasing the uptake of therapeutic antibodies in the brain (Yu et al, 2011). The LDLR family represents a group of cell surface receptors that binds apolipoprotein (Apo) complexes (lipid carriers) for the internalization into the lysosomes (Boren et al, 1998; Brown & Goldstein, 1986; Stefansson et al, 1995). On the surface of the BBB, the binding of LDLR to Apo results in transcytosis on the abluminal side of the BBB, where Apo is released and subsequently taken up by neurons and astrocytes. Fusing the LDLR-binding domain (BD) of ApoB (one of the major types of Apo) to hydrolytic enzymes has been shown to result in an efficient delivery of chimeric enzymes to the CNS (Spencer & Verma, 2007; Spencer et al, 2011).

In this study, we developed and tested a novel minimally invasive approach for the treatment of the brain pathology in MPS IIIA. This strategy makes use of somatic gene transfer based on intravenous injections of adeno-associated vector serotype 8 (AAV2/8) in order to selectively transduce the liver with a modified copy of a sulphamidase gene containing the LDLR binding domain of the ApoB at the C-terminal. Additionally, in order to increase sulphamidase secretion from the liver and thus the amount of the enzyme available in the blood stream, the modified sulphamidase was also engineered with an alternative signal peptide that belongs to the highly secreted sulphatase, iduronate-2-sulphatase (IDS; Polito & Cosma, 2009).

## RESULTS

### Construction and validation of modified sulphamidase *in vitro*

**Figure 1:**
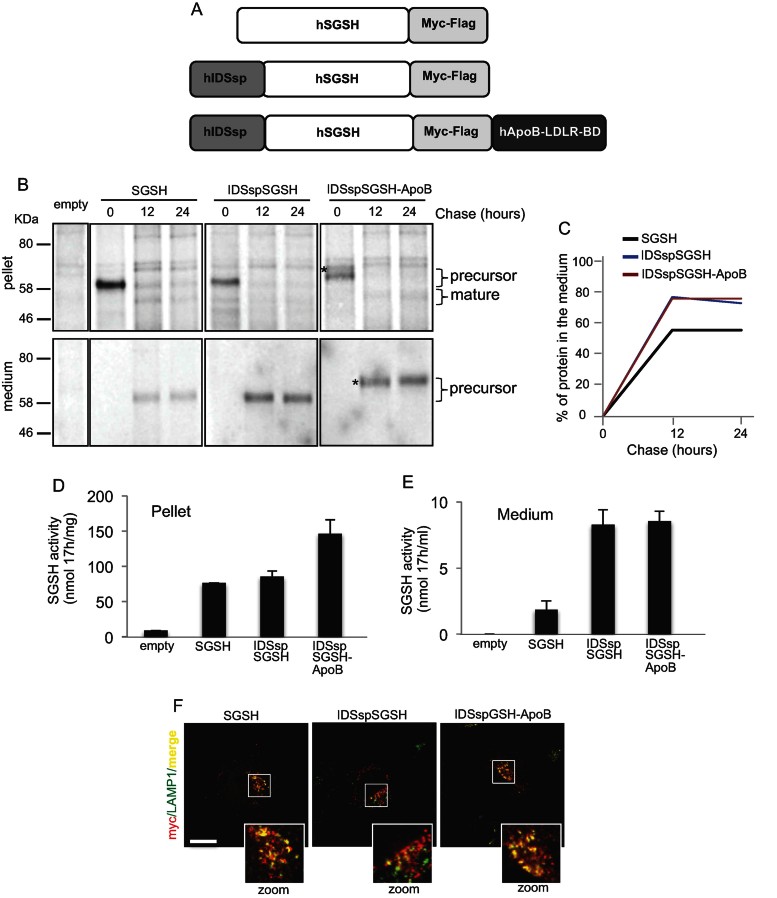
Generation and validation of modified sulphamidase *in vitro*. A. Schematic drawing of the different sulphamidase constructs (wild-type, partially modified and fully modified); see text and Methods section for details. B. MEFs derived from MPS-IIIA mice were transfected with wild-type, partially modified or fully modified sulphamidase encoding plasmids and with an empty vector as control. After 18 h from the transfection the cells were pulsed for 30 min and chased for the indicated times. Anti-flag antibodies were used to immunoprecipitate the flag tagged sulphamidase enzymes in cell pellets and conditioned medium before autoradiography. The asterisk indicated the precursor form detected in the cell pellet and conditioned medium of cells transfected with IDSspSGSH-ApoB. C. The percentage of secreted labelled proteins in the medium (amount of protein in the medium *vs.* amount of protein in both medium and cell pellet) for each SGSH construct was calculated from the densitometric quantification of the bands. D,E. MPS-IIIA MEFs were transfected as indicated. Pellet (D) and 24 h conditioned media (E) were collected and assayed for sulphamidase activity. The SGSH activity was expressed as nanomoles (nmol) of enzyme normalized against incubation time (17 h) and either mg protein content (pellet activity) or ml of medium (medium activity). Values are means ± SEM of triplicate experiments (D,E); *p < 0.05, Student's *t*-test (D,E). F. Confocal analysis with anti-myc (sulphamidase constructs) and anti-LAMP1 (lysosomes) was performed in transfected MPS-IIIA MEFs. See also enlarged images showing the extent of co-localization (merge in yellow). Scale bar, 10 µm.

In order to generate a modified sulphamidase that was capable of crossing the BBB and targeting the CNS via receptor-mediated transcytosis, we engineered the sulphamidase gene by adding to its C-terminal a coding sequence for LDLR-BD of the apolipoprotein B (ApoB LDLR-BD; Fig 1A). Additionally, in order to increase the secretion of sulphamidase from the liver (the vector target organ), we further engineered the sulphamidase by replacing its own signal peptide (sp) with that of the IDS, a highly secreted sulphatase. The final engineered sulphamidase construct (referred to as “fully modified” sulphamidase) comprised the ApoB LDLR-BD at the C-terminal and the IDS sp at the N-terminal (IDSspSGSH-ApoB; Fig 1A). A partially modified sulphamidase that only contained the alternative signal peptide of the IDS (IDSspSGSH) was also generated (Fig 1A; referred to as “partially modified” sulphamidase). All constructs contained a myc-Flag tag on their C-terminal (upstream to the ApoB-BD in the fully modified sulphamidase; Fig 1A).

To analyse the maturation and secretion of the modified sulphamidase enzymes, we performed pulse&chase experiments on mouse embryonic fibroblasts (MEF) cell lines derived from MPS-IIIA mice (Bhattacharyya et al, 2001; Bhaumik et al, 1999). MPS-IIIA MEFs were transfected with plasmids expressing the sulphamidase coding sequences under the control of a CMV promoter. Controls included cells transfected with a wild-type (unmodified) sulphamidase coding sequence and an empty vector (Fig 1B). After 18 h from the transfection the cells were pulsed for 30 min and chased for 0, 12 and 24 h. Anti-flag antibody was used to immunoprecipitate the flag tagged sulphamidase enzymes in collected both cell pellets and conditioned media. As shown in Fig 1B the precursor forms of wild-type and partially modified SGSH could be detected in the cell pellet as proteins with a molecular mass of approximately 62 KDa. After 12 and 24 h of chase these enzymes were processed in their corresponding mature forms (≈56 kDa). The addition of the ApoB-BD resulted in a slight increase in the size (≈3 KDa shift) of the band corresponding to the fully modified enzyme (Fig 1B upper panels). An additional band with a molecular mass greater than that we expected by the addition of the ApoB-BD was detected at ≈70 kDa (indicated by the asterisk in the Fig 1B). This band could correspond to a precursor form of the ApoB-BD-modified SGSH with a different glycosylation pattern (this explaining the observed further increase in the size). However, this conclusion should be confirmed by more specific analyses. From 12 h of chase the precursor forms of all SGSH enzymes started to appear in the medium (Fig 1B bottom panels). Notably, the ApoB-BD-modified SGSH was mainly secreted as the 70 kDa protein consistently with the hypothesis that this protein corresponds to a precursor form of the fully modified SGSH (Fig 1B bottom panels). The percentage of secreted proteins at each chase time demonstrated that the addition of the IDS sp led to an increase in the secretion rate of the modified sulphamidase enzyme compared to wild-type sulphamidase (Fig 1C). Moreover, the presence of the ApoB-BD C-terminal modification did not affect the increase in the secretion efficiency of the fully modified sulphamidase (Fig 1C). The evaluation of the enzymatic activity in both the pellet and the medium of transfected cells further supported the conclusion that the IDS sp replacement strongly increased the secretion rate of ISDsp-modified sulphamidase compared to wild-type sulphamidase (Fig 1D and E).

Next, we analysed the localization of the modified sulphamidase enzymes in transfected cells. Immunostaining with both anti-myc and anti-LAMP1 antibodies showed a lysosomal localization for both partially and fully modified constructs that was similar to that observed in cells transfected with wild-type sulphamidase (Fig 1F).

### Cross-correction capability of modified sulphamidase enzymes

Cross-correction by lysosomal enzymes is an essential biological process that relies on the M6PR-mediated uptake of lysosomal secreted enzymes, such as sulphamidase, by other cells. The therapeutic approach used in this study was based on converting the liver into a factory organ of ApoB-BD-modified sulphamidase, which is then secreted into the blood stream to be taken up by LDL receptors on the BBB. Therefore, we analysed the cross correction capability and the involvement of different receptors (M6PRs and LDLRs) in the uptake of the fully modified sulphamidase enzyme by using the hepatic cell line (Hepa cells) as factory cells and MPS-IIIA MEF cells as recipient cells. Hepa cells were transfected with plasmids encoding for either modified sulphamidase (partially and fully modified) or wild-type sulphamidase under the control of the liver specific promoter, TBG. Cells were also transfected with an empty vector as control. After 24 h from transfection conditioned media (with a SGSH activity of 20 nmol/17 h/ml) were incubated with MPS-IIIA MEFs for 24 h. The secreted sulphamidase enzyme present in the media derived from cells transfected with either modified (fully and partially modified) or wild-type sulphamidase were efficiently taken up by recipient cells (Table 1). The addition of M6P in the conditioned media derived from cells transfected with either wild-type or partially modified sulphamidase inhibited the uptake of the secreted enzyme by approximately 70% (Table 1). Instead, the presence of M6P inhibited the uptake of ApoB-BD-modified sulphamidase by only 30% (Table 1).

**Table 1:** Sulphamidase uptake in MPS-IIIA MEFs

Transfection In Hepa cells	Empty	SGSH		IDSspSGSH		IDSspSGSH-ApoB	
M6P in conditioned medium	−	−	+	−	+	−	+
Activity in recipient MPS-IIIA MEFs (nmol/17 h/mg)	1.39 (±0.14)	18.05 (±0.18)	5.59 (±0.50)	16.85 (±0.55)	5.40 (±0.63)	16.07 (±0.44)	11.15 (±0.65)

Sulphamidase activities (expressed as nmol/17 h/mg) were measured in MPS-IIIA MEFs cells grown for 24 h with conditioned media (containing 20 nmol/17 h/ml of sulphamidase) derived from Hepa transfected with wild-type, partially modified or fully modified sulphamidase encoding plasmids. As control MPS-IIIA MEFs were also incubated with conditioned medium derived from Hepa cells transfected with an empty vector. Conditioned media were incubated with MEFs in absence or in presence of M6P (5 mM). Values are means ± SEM of triplicate experiments.

These data demonstrated that the ApoB-BD-modified sulphamidase is taken up through a process that only partially depends on the M6P receptors, thus indicating the important involvement of LDL receptors pathway in the uptake process.

**Figure 2:**
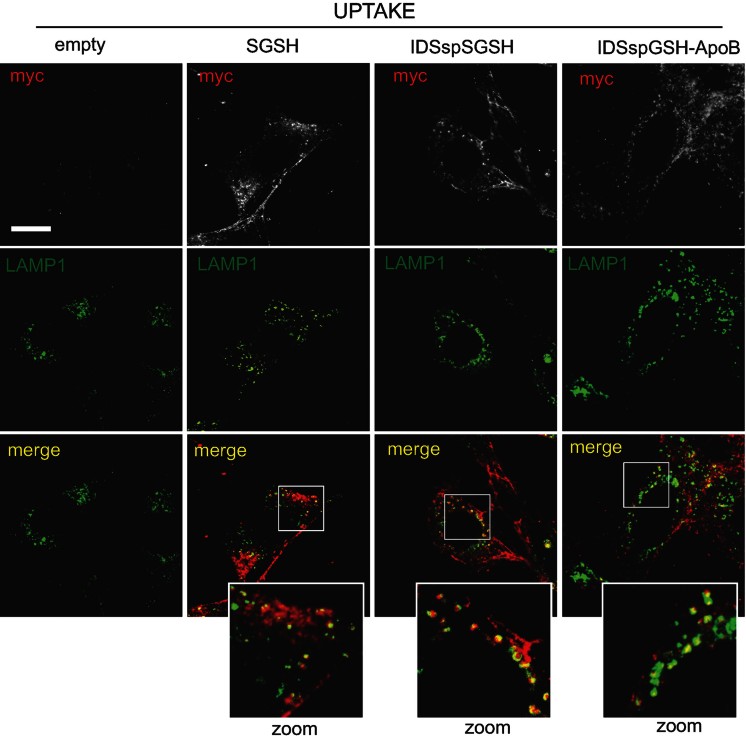
Subcellular localization of modified sulphamidase enzymes upon uptake in cultured cells. Confocal analysis with anti-myc and anti-LAMP1 antibodies was performed on MPS-IIIA MEFs grown for 24 h with conditioned media derived from Hepa transfected with wild-type, partially modified or fully modified sulphamidase encoding plasmids and with an empty vector as control. Enlarged images show the extent of co-localization (merge in yellow). Scale bar, 10 µm.

In order to evaluate the lysosomal re-localization capability of sulphamidase enzymes upon uptake we performed confocal analysis on recipient MPS-IIIA MEF cells. Immunostaining with both anti-myc and anti-LAMP1 antibodies showed that upon uptake, the modified sulphamidase enzymes reached the lysosomal compartment (Fig. 2).

In conclusion, these results showed that the modifications introduced in the sulphamidase enzyme did not affect its specific enzymatic activity or its correct lysosomal localization. The IDS sp replacement allowed the secretion of a greater amount of enzyme. Moreover, secretion efficiency remained unaffected by the ApoB-BD modification. Finally, we demonstrated that recipient cells efficiently took up the secreted form of the modified sulphamidase and that ApoB-BD was effectively implicated in the uptake process of the fully modified sulphamidase.

### AAV2/8-mediated intravascular injection of modified sulphamidase in adult MPS-IIIA mice results in efficient liver transduction and high and stable circulating enzymatic activity

Next, we tested the therapeutic efficacy of the modified sulphamidase enzymes in ameliorating brain pathology in MPS-IIIA mice upon systemic administration using AAV2/8-TBG vectors. AAV2/8 vectors encoding for the fully modified sulphamidase, the partially modified sulphamidase, or the wild-type sulphamidase under the control of the TBG promoter were intravenously delivered in 1-month-old MPS-IIIA mice via retro-orbital injection. As controls, we injected heterozygous (sgsh^+/−^; phenotypically normal; hereafter we refer to these mice as normal mice) and MPS-IIIA mice with AAV2/8-TBG encoding for the green fluorescent protein (GFP). The mice were then analysed at different time points after injection.

**Figure 3:**
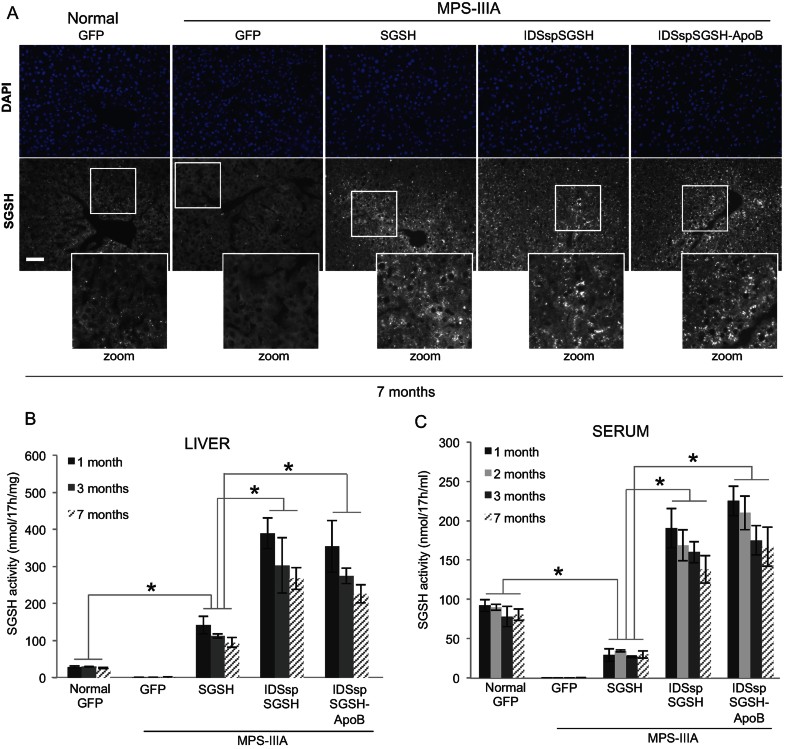
Liver transduction and serum activity in treated mice. A. Immunostaining anti-SGSH (gray) on liver sections derived from MPS-IIIA and normal mice intravascularly injected (at 1 month of age) with AAV2/8 vectors encoding the indicated sulphamidase constructs under a TBG promoter. The extent of SGSH staining in the injected mice is evident in the enlarged images. Nuclei are stained in blue. Scale bar, 30 µm. B. Total sulphamidase activity in the liver of the indicated experimental group of mice. Sulphamidase activity was assayed at 1, 3 and 7 months after injection and was expressed as nmols (enzymes)/17 h (incubation time)/mg (protein). C. Sulphamidase activity was assayed in the serum of mice at 1, 2, 3 and 7 months after injection and was expressed as nmols (enzymes)/17 h(incubation time)/ml (serum). Each bar in B and C represents the average of 5 injected mice. Values are means ± SEM; *p < 0.05, Student's *t*-test (normal GFP *vs.* MPS-IIIA SGSH, MPS-IIIA SGSH *vs.* MPS-IIIA IDSspSGSH and MPS-IIIA SGSH *vs.* MPS-IIIA IDSspSGSH-ApoB for each time-point).

Immunostaining analysis and the measurement of enzymatic activity showed that the liver was efficiently transduced upon AAV2/8-TBG-mediated injection of the modified and wild-type sulphamidase enzyme (Fig 3A and B). The total enzymatic activity in the liver was high and sustained up to 7 months after injection (Fig 3B). The correction of sulphamidase activity in the liver of treated MPS-IIIA mice also led to a recovery of normal liver mass (Supporting Information Fig S1A) and normal mean body weight (Supporting Information Fig S1B) in both female and male MPS-IIIA mice.

Wild-type sulphamidase enzyme is poorly secreted from the liver (Fig 3C). Secretion efficiency is essential in order to determine the amount of circulating sulphamidase potentially available to target the brain. The sulphamidase activity in the serum of MPS-IIIA mice expressing (in the liver) the IDSsp-modified sulphamidase enzymes (*i.e.* partially and fully modified sulphamidase) was 7–9 times higher than the enzymatic activity measured in the MPS-IIIA mice expressing the wild-type sulphamidase (Fig 3C). This build-up reflected a significant increase in the secretion rate of the modified sulphamidase due to the IDS sp replacement. Importantly, the enzymatic activity in the serum of MPS-IIIA mice treated with modified sulphamidase was sustained up to 7 months after injection, thus indicating that our approach resulted in stable liver sulphamidase expression and secretion (Fig 3C).

### ApoB-BD modification allows sulphamidase BBB crossing in treated MPS-IIIA mice

**Figure 4:**
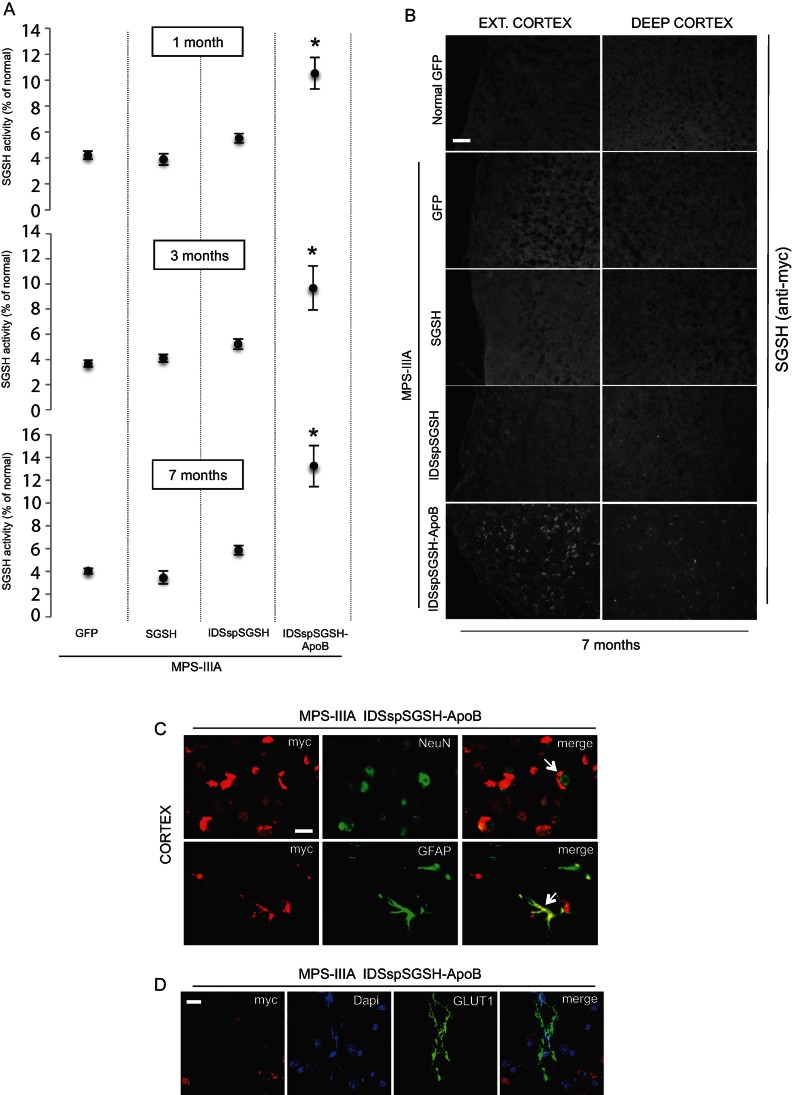
ApoB-BD allows BBB crossing and brain delivery in treated MPS-IIIA mice. A. Sulphamidase activity was assayed in the total brain of MPS-IIIA at 1, 3 and 7 months after injection of the indicated constructs. Sulphamidase activity was expressed as the percentage of the enzyme activity measured in control normal mice. At least five mice were assayed for each experimental group at each time-point. Values are means ± SEM. *p < 0.05, Student's *t*-test (MPS-IIIA IDSspSGSH-ApoB *vs.* MPS-IIIA GFP for each time-point). B. Brain cryosections from the indicated experimental group of mice were stained with anti-myc antibodies to assess sulphamidase brain delivery. Scale bar, 30 µm. C. Brain cryosections from AAV2/8-TBG-IDSspSGSH-ApoB injected MPS-IIIA mice were stained for both myc (red) and either NeuN (green) or GFAP (green). In the upper panel, the arrow indicated a NeuN-positive cell expressing the fully modified SGSH (stained with myc). In the bottom panel the arrow indicated a GFAP-positive cell expressing the fully modified SGSH (stained with myc). D. Brain cryosections from AAV2/8-TBG-IDSspSGSH-ApoB injected MPS-IIIA mice were stained for both myc (red) and GLUT1 (green). DAPI stained nuclei (blue). Scale bar 10 µm (C,D).

In order to evaluate the efficiency of BBB crossing and CNS uptake in treated MPS-IIIA mice we measured sulphamidase activity in total brain samples. As expected, control AAV-GFP-injected MPS-IIIA mice exhibited 3–5% of the enzymatic activity measured in normal AAV-GFP-injected mice (Fig 4A). MPS-IIIA mice expressing either wild-type or partially modified sulphamidase did not show any significant increase in brain enzymatic activity (Fig 4A). In contrast, the AAV2/8-TBG-mediated injection of the fully modified sulphamidase in MPS-IIIA mice led to a significant increase in sulphamidase activity levels up to 10–15% greater than the activity measured in the brain of control mice (Fig 4A). The increase in sulphamidase activity observed in the brain of MPS-IIIA mice expressing the fully modified sulphamidase was also associated with a specific anti-myc immunostaining signal, as revealed by immunofluorescence analysis (Fig 4B). This signal co-localized with both the neuronal marker (NeuN) and the astroglia marker (GFAP), which indicated that the fully modified sulphamidase was taken up by both neuronal and glia cells (Fig 4C). In addition, immunofluorescence with anti-myc and anti-GLUT1 (a marker of BBB endothelial cells) on the brain sections of mice expressing the fully modified sulphamidase revealed the presence of the modified enzyme outside the endothelial cells of the BBB (abluminal side) (Fig 4D). Consistent with the results obtained in the enzymatic assays, the anti-myc specific signal was absent in the brain of MPS-IIIA mice expressing either wild-type or partially modified sulphamidase (Fig 4B).

These data demonstrated the capability of the fully modified sulphamidase enzyme to cross the BBB and to be taken up by brain cells of MPS-IIIA mice upon secretion from the liver. Moreover, these data showed that this capability is specifically due to the presence of ApoB-BD at the C-terminal of the modified sulphamidase.

### Brain pathology is corrected in MPS-IIIA treated with fully modified sulphamidase

**Figure 5:**
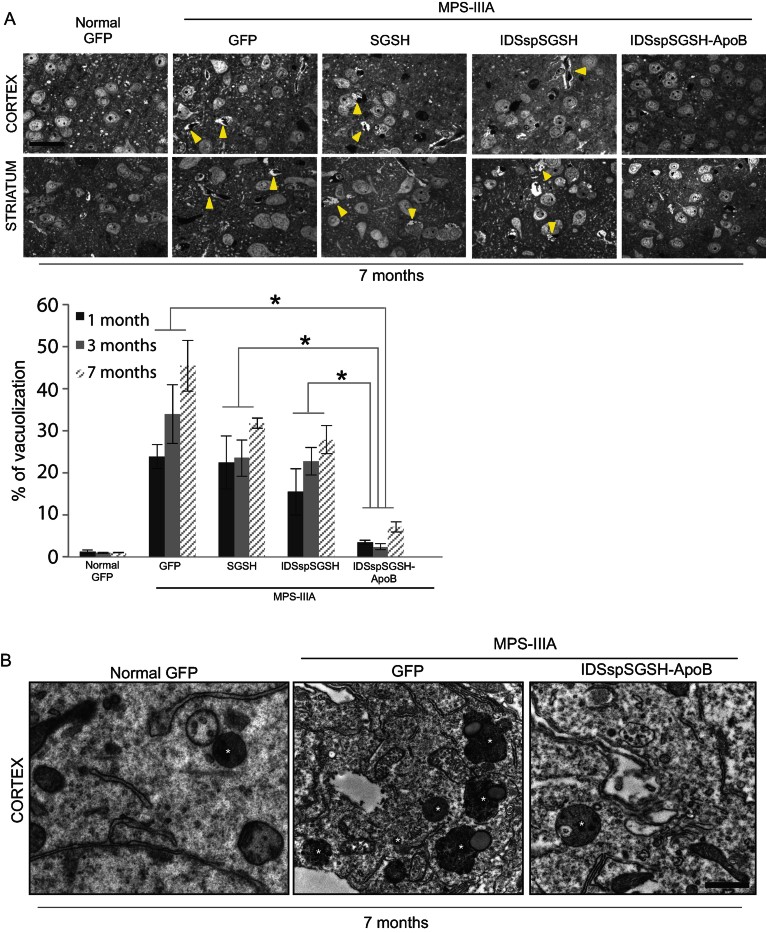
Pathological vacuolization in the brain of treated MPS-IIIA mice. A. Ultra-thin brain sections from the indicated experimental group of mice (7 months after the injection) were stained with toluidine blue to evaluate the extent of vacuolization (yellow arrowheads indicate extensively vacuolated cells). The graph below shows a semi-quantitative analysis of vacuolated cells generated by analysing 100 cells for each experimental group of mice at 1, 3 and 7 months after injection. Each bar represents the average of 3 injected mice (values are means ± SEM). *p < 0.05 Student's *t*-test (normal GFP *vs.* MPS-IIIA SGSH, MPS-IIIA SGSH *vs.* MPS-IIIA IDSspSGSH and MPS-IIIA SGSH *vs.* MPS-IIIA IDSspSGSH-ApoB for each time-point). Scale bar, 30 µm. B. Cortex brain regions were fixed and prepared for EM as described in Materials and Methods. Asterisks in each image indicate the lysosomes. Panels on the left show lysosomes in the brain of control normal mice. MPS-IIIA cortex exhibits numerous large lysosome-like structures. Cortex from MPS-IIIA mice treated with AAV2/8-TBG containing the IDSspSGSH-ApoB-BD shows a reduced number of enlarged lysosomes. Scale bar 1 µm.

Next, we evaluated the correction of the pathological phenotype in the brain of injected MPS-IIIA mice. A time-dependent increase in the pathological vacuolization was observed in the brain of MPS-IIIA mice when compared to control normal mice, as determined by toluidine blue staining (Fig 5A and Supporting Information Fig S2). The injection of fully modified sulphamidase in MPS-IIIA mice led to a significant rescue of pathological vacuolization at all time points analysed (Fig 5A and Supporting Information Fig S2). Electron Microscopy analysis of the brain cortex confirmed a reduced amount of storage vacuoles in the brain of treated MPS-IIIA mice when compared to control MPS-IIIA mice (Fig 5B). The intravascular injection of either wild-type or partially modified sulphamidase in MPS-IIIA mice did not result in an improvement of pathological vacuolization in the affected brain, which was consistent with the observed absence of significant levels of sulphamidase in the brain (Fig 5A and Supporting Information Fig S2).

Vacuolization is a measure of glycosaminoglycans (GAGs) storage in the brain of affected MPS-IIIA mice. As expected, GAG levels were significantly higher in the brain of control MPS-IIIA mice compared to control normal mice (Supporting Information Fig S3). Moreover, in accordance with the reduced pathological vacuolization (Fig 5A), we observed a significant decrease in accumulated GAGs only in the brain of MPS-IIIA mice expressing the fully modified sulphamidase (Supporting Information Fig S3).

**Figure 6:**
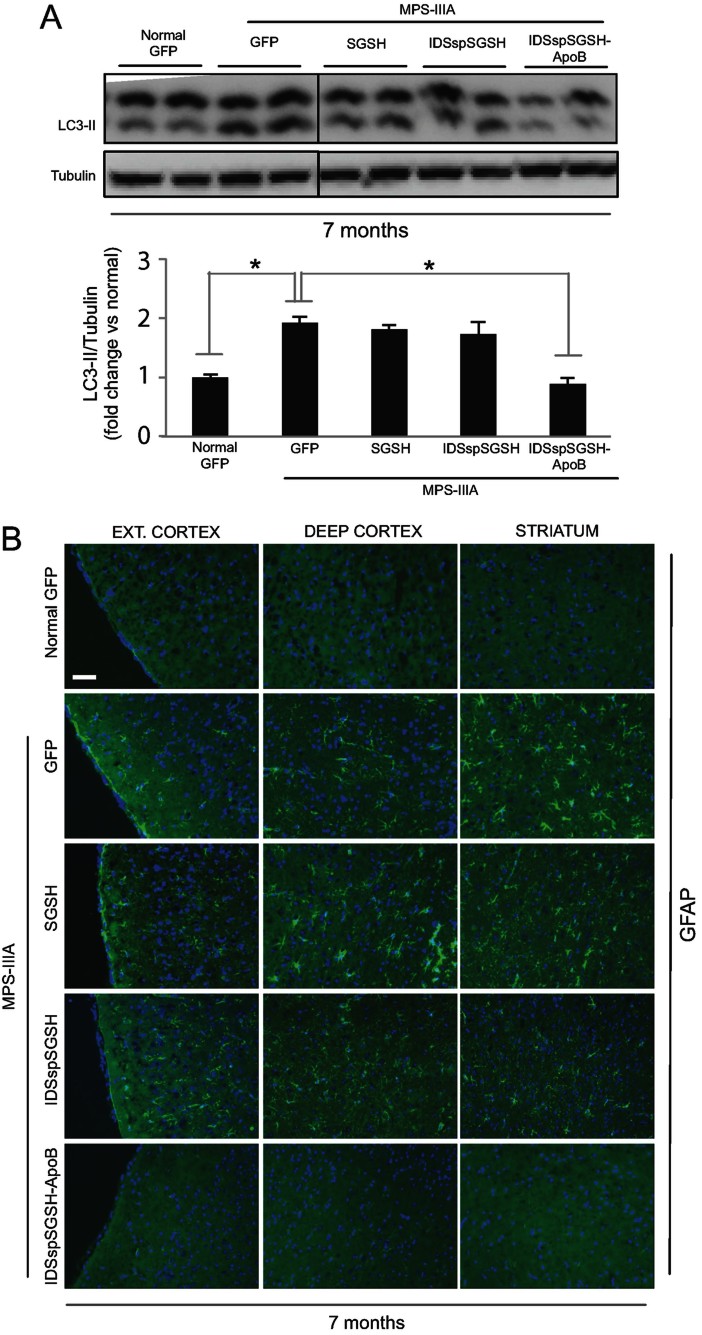
Brain pathology in treated MPS-IIIA mice. A. Autophagy flux was analysed in the brain of the indicated experimental group of mice by ant-LC3 Western blot on total brain lysates. Two mice from each experimental group are shown. A densitometric quantification of the bands was shown in the graph below. Values are means ± SEM (triplicate experiments); *p < 0.05, Student's *t*-test (normal GFP *vs.* MPS-IIIA SGSH, MPS-IIIA SGSH *vs.* MPS-IIIA IDSspSGSH and MPS-IIIA SGSH *vs.* MPS-IIIA IDSspSGSH-ApoB). B. Brain cryosections from the indicated experimental group of mice were immune-stained with anti-GFAP (green) to evaluate the presence of astrogliosis in the brain of treated mice. DAPI (blue) stains nuclei. Scale bar, 30 µm.

The autophagic pathway is impaired in MPS-IIIA mice due to lysosomal accumulation and a consequent block of fusion between lysosomes and autophagosomes (Fraldi et al, 2010; Settembre et al, 2008). This becomes evident in affected mice at 6–7 months of age when brain pathology is fully established. To evaluate whether the rescue of pathological vacuolization was also associated with a normalization of the autophagic flux, we compared the levels of LC3-II in the brain of control and treated MPS-IIIA mice at 7 months after injection. As previously observed, LC3-II levels were significantly greater in the brain of MPS-IIIA mice than in the brain of control normal mice (Fig 6A). The injection of wild-type or partially modified sulphamidase did not decrease the levels of LC3-II; however, the injection of fully modified sulphamidase led to the normalization of LC3-II levels (Fig 6A).

Finally, we analysed the presence of pathological signs of inflammation in the brain of injected MPS-IIIA mice. We observed a severe astrogliosis (evidenced by GFAP signal) along with an increased activated microglia (evidenced by IBA-I signal) in the brain of MPS-IIIA mice compared to control mice (Fig 6B and Supporting Information Fig S4). Instead, the brain of MPS-IIIA mice expressing the fully modified sulphamidase displayed no signs of pathological inflammation (Fig 6B and Supporting Information Fig S4). Conversely, no significant reduction in GFAP staining was detected in the MPS-IIIA mice that were injected with AAVs encoding wild-type or partially modified sulphamidase (Fig 6B).

### Improved behavioural phenotype in MPS-IIIA mice treated with fully modified sulphamidase

**Figure 7:**
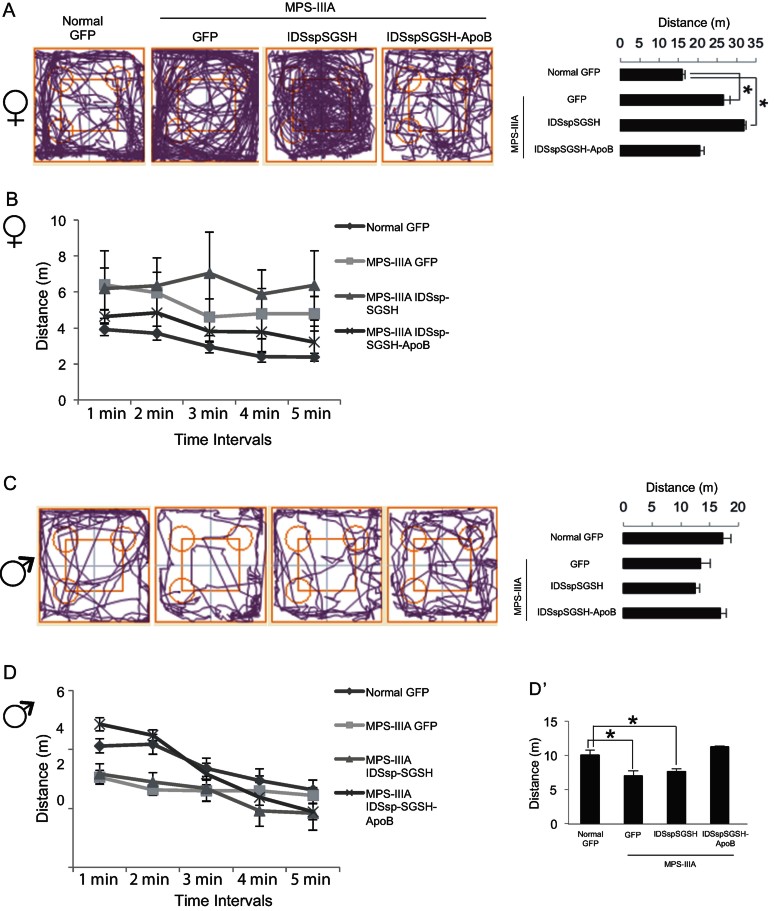
Open field path length in treated MPS-IIIA mice. A–D. Track plots and means total distance (m) travelled by female (A) and male mice (C) (treated as indicated) during 5 min in the open field. Mean distance travelled (m) minute by minute over 5 min in female (B) and male (D) treated mice. (D′) Mean total distance travelled (m) by male treated mice during the first two and half minutes in the open field. Values are means ± SEM; *p < 0.05 (*vs.* normal GFP), Bonferroni-Dunnet *post hoc* test. See Methods section for details on behavioural procedures.

To determine whether the amelioration of CNS pathology in MPS-IIIA mice that were injected with AAVs encoding the fully modified sulphamidase resulted in an improvement in the behavioural phenotype, we monitored the exploratory activity in control normal mice, control MPS-IIIA mice, and MPS-IIIA mice treated with fully modified sulphamidase. In addition, to analyse the impact of ApoB-BD modification we also compared the behavioural exploratory phenotype of MPS-IIIA mice treated with fully modified sulphamidase with that of MPS-IIIA mice treated with partially modified sulphamidase. Exploratory activity was monitored by measuring the path the mice travelled in an open field during 5-minute sessions. We found a significant increase in path length in female MPS-IIIA mice compared to their control littermates (Fig 7A and B). In contrast, male MPS-IIIA mice travelled less distance than their control counterparts (Fig 7C), thus revealing a significant hypoactivity of male mice. This result became more evident when distance travelled was analysed during the first minutes of the task (Fig 7D and D′). Indeed, because animals tend to be especially active during initial exposure to a novel environment, the first minutes of observation represent the best period in which to discern any reduction in the novelty-directed exploratory behaviour. All together, these results were consistent with previous findings, which show that MPS-IIIA female mice are hyperactive in the open field (Crawley et al, 2006; Langford-Smith et al, 2011), while male mice exhibit reduced exploratory activity (Hemsley & Hopwood, 2005; Lau et al, 2010). Hyperactivity was selectively reduced in female MPS-IIIA mice expressing the fully modified sulphamidase, but not in those expressing the partially modified sulphamidase (Fig 7A and B). Similarly, MPS-IIIA male mice expressing the fully modified sulphamidase showed an improvement in the hypoactive phenotype, but those expressing the partially modified sulphamidase did not (Fig 7C, D and D′).

Taken together, these data demonstrated that the level of sulphamidase activity achieved in the brain of MPS-IIIA mice that were injected with AAVs encoding the fully modified sulphamidase is sufficient to improve behavioural abnormalities.

## DISCUSSION

In this study, we demonstrated the therapeutical efficacy of a novel, minimally invasive gene therapy approach to treat CNS lesions in MPS-IIIA mice. This therapeutic strategy is based on liver-directed delivery of a highly secreted form of the sulphamidase enzyme, which was modified to be actively transported across the BBB in order to be efficiently taken up by brain cells. The intravenous injection was used as a minimally invasive method of administration to target the liver, while high enzyme secretion and receptor-mediated transcytosis were used as efficient ways to increase circulating enzyme and overcome the BBB. Moreover, treating mice at an adult stage allowed us to demonstrate the therapeutic efficacy of the chimeric enzyme in the adult brain, when the BBB is completely impermeable and the MPS-IIIA pathological phenotype is fully established. Several viral-mediated delivery approaches can be used to systemically target the liver with a gene of interest. The most effective for therapeutic approaches are those based on helper-dependent adenovirus (HDAd) or on AAV vectors with high liver tropism (serotype 2/8) (Brunetti-Pierri & Ng, 2011; Mingozzi & High, 2011). AAV2/8 vectors are safe, allow high-levels of transgene expression and have minimal immunostimulatory properties, and therefore represent the most promising vectors to use in human liver-directed gene therapy protocols (Nathwani et al, 2011). Based on these considerations we chose AAV2/8 as a vehicle to deliver the modified sulphamidase to the liver. The efficacy of this approach is also dependent on the ability of the liver to efficiently secrete the modified sulphamidase in the blood stream, in order to maintain high and stable levels of circulating enzyme available to reach the brain. In fact, serum levels of the therapeutic enzyme may represent a critical factor in the treatment of the brain for disorders that are due to an enzyme deficiency (Grubb et al, 2008; Rozaklis et al, 2011). Our data demonstrated that although the liver is efficiently transduced by wild-type sulphamidase upon AAV2/8-mediated delivery, the enzyme is poorly secreted (Fig 3A and C). The replacement of the sulphamidase sp with that belonging to IDS strongly increased the secretion efficiency thus building up the amount of serum activity 7–9 times with respect to the activity found in MPS-IIIA mice expressing the wild-type sulphamidase (Fig 3C). Notably, the differences in the serum levels of sulphamidase activity between MPS-IIIA mice expressing different SGSH constructs and control normal mice were lower than those observed in the liver (Fig 3A and C). This may be due to the fact that the exogenous sulphamidase secreted from the liver of MPS-IIIA mice injected with AAVs encoding different SGSH constructs can be partially cleared due to its uptake by ‘off target’ organs (which lack functional and active sulphamidase) and by the liver itself. This clearance is not effective in wild-type mice in which all the organs, including the liver, express the endogenous sulphamidase enzyme. In addition, we did not observe the presence of obvious signs of pathophysiology in different ‘off-targets’ organs such as kidney, spleen, heart and lung indicating the absence of relevant toxic effects. However, we cannot exclude the potential occurrence of some toxic effects, especially at long term.

The rationale behind the use of the IDS sp in order to modify the sulphamidase enzyme is based on the fact that IDS is secreted from the liver at very high level (Polito & Cosma, 2009). However, the replacement of IDS sp alone did not achieve sufficient sulphamidase enzymatic activity to allow the BBB crossing of the sulphamidase enzyme. Indeed, we observed significant brain targeting only when the sulphamidase was additionally modified with the ApoB-BD, indicating that this modification allowed the sulphamidase to effectively cross the BBB. Our results are in agreement with previous data that demonstrate that sulphamidase is normally unable to cross the BBB in adult brain due to a reduction in the expression of M6P receptors on the BBB during development (Gliddon & Hopwood, 2004; Urayama et al, 2008). The crossing of the BBB as a result of the overloading the blood stream with lysosomal enzymes has been demonstrated, however, the mechanisms involved are not well understood (Blanz et al, 2008; Polito & Cosma, 2009; Vogler et al, 2005). Similar results have recently been obtained for sulphamidase by Bosh et collaborators (Ruzo et al, 2012). These authors observed a low but detectable brain sulphamidase activity associated with a partial rescue of CNS phenotype in MPS-IIIA mice that received and intravenous injection of AAV encoding the wild-type sulphamidase (Ruzo et al, 2012). However, in our present study we were unable to detect BBB crossing and brain delivery upon AAV2/8-mediated delivery of wild-type sulphamidase to the liver, even when sulphamidase secretion and serum loading were strongly increased by IDS sp replacement. This discrepancy could be explained in part by the higher doses of AAV vectors used by Bosh et al (a total of 1 × 10^12^ particles in their study *vs.* a total of 1 × 10^11^ particles in ours), which resulted in a serum specific activity that was about 2–3 times greater than the specific activity we measured in mice expressing IDSsp-modified sulphamidase. These data would suggest that higher levels of sulphamidase overloading in the serum than those obtained in our study could result in a partial BBB crossing of sulphamidase. However, it remains still difficult to reconcile this conclusion with recent data obtained by Hemsley et collaborators, which showed that high doses of sulphamidase administered by ERT protocols (*i.e.* 10 and 20 mg/kg compared to conventional doses of 1 mg/kg) do not result in BBB crossing (Rozaklis et al, 2011). Instead, these data support our conclusion that a higher serum dose of sulphamidase alone is not enough to elicit BBB crossing.

In this study, we showed that the ApoB-BD modification allowed effective sulphamidase brain delivery, which led to a complete rescue of brain pathology including associated behavioural abnormalities. However, peripheral organ alterations can also affect the behavioural phenotype. Therefore, the rescue produced by the combination of IDS sp replacement and ApoB-BD addition could be due to either central or peripheral effects of the treatment, or a combination thereof. Nevertheless, if the effect was the result of a peripheral increase in enzyme activity, we should have also found a rescue after treatment with partially modified sulphamidase, but this was not the case. Furthermore, the fact that treatment with fully modified sulphamidase reduced hyperactivity in MPS-IIIA female mice but increased hypoactivity in MPS-IIIA male mice suggests that this treatment is not broadly modulating the general level of activity in MPS-IIIA mice, but that it is specifically modulating the neuropathological mechanism underlying the gender specific phenotype of MPS-IIIA.

In this study, we observed a variability in brain delivery following treatment with the fully modified sulphamidase. This variability indicates that besides the efficiency of ApoB-BD-mediated BBB transcytosis, additional factors may limit BBB crossing and/or brain delivery of ApoB-BD-modified sulphamidase. A possible explanation for such variability could be the presence of dissimilarities in the BBB molecular signatures of the mice used in the study. Indeed, the BBB molecular signature, which determines transcytosis, may change significantly in pathological conditions and in response to different metabolic conditions (Chen et al, 2009). Specifically, LDLRs (those mediating the ApoB transcytosis) could be expressed at different levels on the BBB of the experimental mice used. The factors involved in such variability and how these factors potentially contribute to limiting BBB crossing and/or brain delivery of circulating ApoB-BD-modified sulphamidase are questions that should to be investigated in future studies.

To our knowledge, this is the first study that demonstrates that modifying a lysosomal enzyme to both increase liver secretion and to allow BBB receptor-mediated transcytosis may efficiently correct brain pathology in a mouse model of LSD.

The data presented represents a solid proof-of-principle for the development of future clinical approaches based on the AAV2/8-mediated delivery of modified sulphamidase in MPS-IIIA patients. Importantly, it should be stressed that a single administration of AAV2/8 carrying the modified sulphamidase gene was sufficient to achieve a long-term (7 months from injection) correction of the brain pathology. This represents a clinically relevant aspect of the approach that could avoid possible toxic effects caused by repeated injections of high doses of AAV2/8 viral vectors. However, the generation of additional preclinical data on possible long-term toxic side effects of sulphamidase overloading in the blood stream, and on the immunogenic properties of the modified sulphamidase itself could improve the therapeutic efficacy and facilitate the clinical translation of this approach.

Finally, our data raise the possibility of implementing existent ERT protocols for MPS-IIIA and other LSDs with enzymes carrying specific brain targeting peptides. This opens the potential exploration of new therapeutic routes that bypassing the ‘gene therapy’ aspect could expedite the clinical translation of approaches based on the delivery of brain targeting-modified lysosomal enzymes.

## MATERIALS AND METHODS

### Construction of chimeric sulphamidase constructs and AAV vectors

PRC amplification and ligation standard procedures were used to construct chimeric sulphamidase expression cassettes. AAV2/8 viral vectors were produced according protocols established at AAV TIGEM Vector Core. See Supporting Information for details.

### Secretion and uptake studies in cells

MPS-IIIA MEF cells and Hepa were maintained in DMEM supplemented with 10% FBS and penicillin/streptomycin (normal culture medium). Sub-confluent cells were transfected using LipofectamineTM 2000 (Invitrogen) according to manufacturer's protocols. Five hours after transfection the medium was replaced with DMEM 0.5% FBS. For the secretion experiments, the MPS-IIIA MEFs pellet and conditioned media were collected and assayed for enzymatic activity. For uptake experiments, 24 h after medium changing conditioned media derived from transfected Hepa cells and containing sulphamidase activity of 20 nmol/17 h/mL were incubated in absence or in presence of M6P (5 mM final concentration) with sub-confluent MPS-IIIA MEF cells. After 24 h MEFs were harvested by trypsin treatment, washed with PBS by centrifugation (1500*g* for 5 min) and processed for the enzymatic assays. The same cells were also processed for confocal analysis.

### Pulse and chase

MPS-IIIA MEF cells transfected with different chimeric constructs and radiolabelled (pulse) with 30 µCi/10^6^ cells [35S] methionine/cysteine mixture (EasyTag (TM) EXPRE35S35S Protein Labeling Mix, [3S]; PerkinElmer) for 30 min in methionine/cysteine-free medium (Sigma) supplemented with 1% fetal calf serum. Labelling medium was removed and cells were washed with PBS to remove excess free label. Cells were chased with DMEM in the presence of 0.5% fetal calf serum and supplemented with methionine and cysteine. After 0, 12 and 24 h, the chased medium was collected and clarified by centrifugation (1500*g*, 3 min).

Cells were recovered at different time points and lysed on ice for 30 min in 700 µl of 3× flag Lysis buffer (50 mM Tris–HCl pH8, 200 mM NaCl, 1% Triton X100, 1 mM EDTA, 50 mM HEPES). Lysates were clarified by centrifugation and supernatants were immunoprecipitated using agarose-conjugated antibody against flag (anti-flag M2 affinity Gel, A2220 Sigma–Aldrich). After extensive washing with lysis buffer, the immunoprecipitate was subjected to SDS–PAGE followed by autoradiography.

### Immunofluorescence microscopy

Cells were washed three times in cold PBS and then fixed in 4% paraformaldehyde (PFA) for 15 min. Fixed cells were washed four times in cold PBS, permeabilized with blocking solution (0.1% Saponin and 10% FBS in PBS) for 30 min and immuno-labelled with appropriate primary antibody: anti-c-Myc-Cy3 (C6594 Sigma, St. Louis, MO), Polyclonal Rabbit anti LAMP1 (SIGMA). Alexa-fluor secondary antibodies were from Molecular Probe (Invitrogen). Cells were then washed four times in cold PBS and mounted in Vectashield mounting medium. Confocal microscopy was performed by using a Zeiss LSM 510 microscope equipped with a Zeiss confocal-scanning laser using a 63× numerical aperture 1.4 objective.

Medial sagittal sections of frozen brain tissue were cut on a cryostat at either 6 or 30 µm of thickness, fixed with 4% PFA, permeabilized (PBS, 0.2% Tween-20 and 10% foetal bovine serum) and stained with appropriate primary antibodies: Mouse monoclonal anti-c-Myc-Cy3 (C6594 Sigma, St. Louis, MO), rabbit polyclonal anti-GFAP (Dako), mouse monoclonal anti-NeuN (MAB 377 Millipore), rabbit anti-Iba1 (234003 Synaptic Systems), mouse monoclonal anti-GLUT1 (ab-40084 Abcam) and sheep polyclonal anti-hSGSH (a gift from Hopwood's lab), Alexa-fluor secondary antibodies were from Molecular Probe (Invitrogen) Stained sections were mounted with Vectashield (Vector Laboratories, CA, USA). Background signal of anti-myc staining was quenched by using ammonium-chloride 50 mM in PBS solution after PFA fixation. Photographs were taken by an epi-fluorescence microscope. NeuN was visualized in 30 µm-thick frozen brain sections.

### Animals, AAV administration and tissue collection

Homozygous mutant (sgsh^−/−^; phenotypically MPS-IIIA affected) and heterozygous (sgsh^+/−^; phenotypically normal) C57BL/6 mice were used (Bhattacharyya et al, 2001; Bhaumik et al, 1999; Fraldi et al, 2007). Consequently, the term ‘normal mice’ is used to refer to the mouse phenotype and not to the mouse genotype. The AAV vector stocks (1 × 10^11^ particles in 300 µl) were administered via the retro-orbital sinus in MPS-IIIA and control normal mice at 12 weeks of age. After being collected, the serum was harvested and stored frozen until use. To collect liver and brain, mice were perfused with PBS (pH 7.4), and to prepare brain samples for sulphamidase assays standard capillary depletion protocols were used. See Supporting Information for details on tissue collection.

Experiments were conducted in accordance with the guidelines of the Animal Care and Use Committee of Cardarelli Hospital in Naples and authorized by the Italian Ministry of Health.

### SGSH activity

SGSH activity was measured in cells, serum and tissues by a fluorogenic substrate (Moscerdam Substrates, Netherlands) following established protocols (Fraldi et al, 2007).

### Toluidine blue staining and Electron microscopy

Fixed samples of specific brain regions were post-fixed in 1% osmium tetroxide, dehydrated and embedded in resin. One micron sections were stained with 1% toluidine blue and examined by light microscopy. Ultra-thin sections from the selected region of cortex were cut and stained with 0.5% uranile acetate. Morphological analysis of cellular and subcellular structures was carried out at EM TIGEM core.

### Body weight and behavioural procedures

A total number of 27 normal GFP treated, 14 MPS-IIIA GFP-treated, 8 MPS-IIIA IDSspSGSH treated, and 8 MPS-IIIA IDSspSGSH-ApoB mice (males and females) were subjected to the behavioural tasks. Animals were of ages between 8 and 10 months. They were housed in Plexiglas cages (18 × 35 × 12 cm^3^) with free access to food and water and kept at a temperature range between 20 and 23°C. These tests were carried out in a behavioural testing room maintained under constant light, temperature and humidity. The mice were tested during daylight hours (between 9 AM and 6.00 PM). Before testing animals were habituated to the testing room for at least 30 min. All behavioural tests were performed by the same experimenter (EDL).

Body weight was measured twice at the interval of 2 weeks apart; the average between these two measurements was analysed using one-way ANOVA.

During the open field task the MPSIIIA mice were placed in the middle of a plexiglas arena with a masonite base (43 × 32 × 40 cm^3^) placed on a flat surface 70 cm over the floor. Animals were left free to explore the device for 5 min. The distance travelled (m) was recorded for 5 min using a video camera (PANASONIC WV-BP330) hanging over the arena that was connected to a video-tracking system (ANY-MAZE Stoelting-USA). The following elements were monitored each minute: covered distance (m), time spent in the fringe area (s), and time spent in the central area (sec). Mean total distance and the percentage of time spent in the centre of the arena (centre time/600 s) were analysed using one-way ANOVA. The treatment effects were evaluated by comparing the behaviour of treated mice to that of their respective heterozygous (normal) counterparts using the Bonferroni-Dunnet *post hoc* test. Mean distance travelled each minute (time interval) was analysed using a two-way ANOVA for repeated measure (5 levels).

**The paper explained**

**PROBLEM**

Mucopolysaccharidoses (MPS) type IIIA is a lysosomal storage disease (LSD) caused by the body's inability to produce a specific lysosomal enzyme, sulphamidase. In individuals with MPSs, the missing or defective enzyme results in the storage of materials in virtually every cell of the body with consequent damage and cell death. The central nervous system is the predominant target of damage in MPS-IIIA and in most of the MPSs. Gene therapy and ERT represent feasible therapeutic options for the treatment of neurodegenerative LSDs. However, these approaches have been demonstrated to be effective only when the viral vectors or enzymes are supplied directly to the diseased brain. Conversely, they are generally inefficient when viral vectors or enzymes are supplied via an intravascular route due to the obstacle presented by the blood-brain barrier (BBB). Therefore, the available strategies to treat brain lesions associated with LSDs are very invasive protocols.

**RESULTS**

In this study, we developed and tested a minimally invasive therapeutic strategy for the treatment of brain lesions in MPSIIIA. This strategy was based on the use of a chimeric sulphamidase, which was engineered by adding both an alternative signal peptide belonging to the highly secreted sulphatase IDS, and the binding domain of the apolipoprotein B, a protein known to be actively transported across the BBB by receptor-mediated transport. We demonstrated that upon systemic AAV2/8 vectors-mediated liver targeting in MPS-IIIA mice, the chimeric sulphamidase was highly and sustainably secreted from liver and was able to efficiently cross the BBB resulting in efficient brain delivery. Brain delivery led to an effective amelioration of the overall brain pathology and behavioural phenotype in treated MPS-IIIA mice.

**IMPACT**

Our results provide proof-of-concept on the feasibility and efficacy of systemic therapies for the treatment of brain lesions in LSDs that are based on modifying lysosomal enzymes in order to increase their secretion from the liver and to enable crossing of the BBB.

### Data analysis

Data are expressed as mean ± 1 SEM ANOVA (or RMANOVA) and student's *t*-test were used to compare different treatment groups of either mice or cells. A *p*-value of <0.05 was considered to be statistically significant.

## Author contributions

NCS performed research and analysed data; LDO and EN maintained the mouse colony, performed mouse injections and processed tissues; CM performed research; IS contributed by performing co-localization experiments; CS helped in mouse injections; EP performed EM analysis; PS and EDL performed behavioural analysis; AB supervised the research; AF designed research, supervised research and wrote the manuscript.

## For more information

TELETHON-Italy Foundation official website:

http://www.telethon.it

National MPS society official website:

http://www.mpssociety.org

## Notes

As a service to our authors and readers, this journal provides supporting information supplied by the authors. Such materials are peer reviewed and may be re-organized for online delivery, but are not copy-edited or typeset. Technical support issues arising from supporting information (other than missing files) should be addressed to the authors.
