# Supplementary material for: A highly secreted sulphamidase engineered to cross the blood-brain barrier corrects brain lesions of mice with mucopolysaccharidoses type IIIA
Source: EMBO Mol Med. 2013 Apr 9;5(5):675–90. doi: 10.1002/emmm.201202083 (PMC3662312; doi:10.1002/emmm.201202083)
Supplement: Supplementary file 1 [file emmm0005-0675-sd1.pdf]

EMBO Molecular Medicine

A highly secreted sulfamidase engineered to cross the blood-brain barrier corrects brain lesions of mice with mucopolysaccharidoses type IIIA

Nicolina Cristina Sorrentino, Luca D'Orsi, Irene Sambri, Edoardo Nusco, Ciro Monaco, Carmine Spampanato, Elena Polishchuk, Paola Saccone, Elvira De Leonibus, Andrea Ballabio and Alessandro Fraldi

Corresponding author: Alessandro Fraldi, TIGEM

Review timeline:

Submission date:	26 September 2012
Editorial Decision:	20 November 2012
Revision received:	31 January 2013
Editorial Decision:	19 February 2013
Revision received:	20 February 2013
Accepted:	20 February 2013

Transaction Report:

(Note: With the exception of the correction of typographical or spelling errors that could be a source of ambiguity, letters and reports are not edited. The original formatting of letters and referee reports may not be reflected in this compilation.)

Editor: Roberto Buccione

1st Editorial Decision 20 November 2012

Thank you for the submission of your manuscript "A highly secreted sulfamidase engineered to cross the blood-brain barrier corrects the CNS pathology of mice with mucopolysaccharidoses type IIIA ". We have now heard back from the three referees whom we asked to evaluate your manuscript.

While all three Reviewers acknowledge the relevance and interest of your work, they raise a few issues that I would like you to address in a revised version.

Reviewer 1 mentions a number of specific items that relate to the presentation and discussion of data. S/he asks that specific sulfamidase values, rather than relative activities, be shown. Furthermore, appropriate controls are required to show uptake specificity. Reviewer 1 also suggests that sulfamidase uptake data should be provided for other non-neuronal cells and that the discrepancies with previous work by Ruzo et al. are discussed. Finally, Reviewer 1 feels that the claim that ApoB-modified sulfamidase leads to complete rescue of brain pathology is overstated and would benefit from additional experimental evidence along the lines suggested. We strongly encourage developing the study as far as realistically possible in these directions to strengthen your findings.

Reviewer 3 is concerned that the modification of sulfamidase to increase liver secretion might lead to the production of excessive amounts of the enzyme, and asks the Authors to consider this issue.

S/he also has some concerns regarding figure quality and appropriate use of controls. Reviewer 3 also suggests that to stain activated microglia for IBA-1 would further validate that sulfamidase delivery and GFAP decrease are occurring at the same time.

Finally, all three Reviewers, including Reviewer 2, have a number of very important suggestions to improve interpretation of your data, impact and readability of your work and to enhance its translational potential.

While publication of the paper cannot be considered at this stage, I invite you to submit a revised manuscript addressing the Reviewers' concerns with additional experimental data where appropriate.

Please note that it is EMBO Molecular Medicine policy to allow a single round of revision only and that, therefore, acceptance or rejection of the manuscript will depend on the completeness of your responses included in the next, final version of the manuscript.

We look forward to receiving your revised manuscript as soon as possible. Please find below, together with the Reviewers' comments, important instructions for sumbission of your revision.

***** Reviewer's comments *****

Referee #1 (Comments on Novelty/Model System):

This is a very important manuscript reporting on a novel gene therapeutic approach for enzyme delivery across the blood-brain-barrier. The data have a high impact also for other lysosomal storage disorders affecting the brain. However, the presentation of the data is not satisfying and need to be discussed in more detail. In particular the description and performance of several experiments are incomplete or not precise enough to accept the manuscript in its present form. Several data in various parts of the Figures are sparely described or commented, and the presentation of the references reflect a carelessness to check all parts of the submitted manuscript. The high impact of the Journal requires additional and more detailed data analysis and therefore major revisions. My proposed additional experiments are feasible and not very time consuming but would substantially improve the manuscript.

Referee #1 (General Remarks):

This is a very interesting and exciting manuscript in which Fraldi and colleagues described for the first time an efficient and minimal invasive gene therapeutic approach to target sulfamidase to the brain of sulfamidase-defective MPSIIIA mice. They generated a modified AAV 2/8 that allow the transduction of the liver and the expression of a sulfamidase construct exhibiting an increased secretion capability and a C-terminal ApoB domain for LDLR-mediated transcytosis of the BBB. The study is a logic continuation of the previous report on early intraventricular injection of SGSH in MPSIIIA mice resulting, however, in a higher long-lasting efficacy which might be applicable to other lysosomal storage disorders affecting the brain.
The presentation and discussion of the data, however, require substantial improvement and more precise and informative data sets before publication. In general, the authors should describe all parts of the different Figures in more detail and not only those which are confirmatory.

Major concerns and specific comments:
-Fig 1B: indicate the molecular masses and describe the expected masses of sulfamidase constructs; explain differences between cellular and secreted forms. It is unclear whether or not the cellular forms are processed in the lysosomes. A single blot only will allow the comparison of the different forms but is still misleading to determine the secretion rate. The advantage of the IDSsp on secretion is not obvious and requires pulse-chase experiments.
-Results: The authors should provide specific sulfamidase activity values for the pellet and medium rather than relative activity (provide fold-increases in activity, distribution and uptake for all conditions
-The description of the experimental setup shown in Fig. 1E is rather cryptic. Controls in the presence of M6P or LDL are missing to show the specificity of uptake and the dominance of individual targeting signals in SGSH-ApoB. How much sulfamidase (mU) was offered? According to the Figure legend and the text in the Results part the authors offered equal amounts of conditioned

media to Hepa cells which could explain the increase in uptake of modified SGSH, or why should the uptake of IDSsp SGSH (after loss of the signal peptide in the ER) be significantly (3-fold) higher than wild-type SGSH?
-The authors should provide data on the uptake of sulfamidase in other non-neuronal cells (Fig. 3C) because the MPS pathology has been reported to be more evident in perineuronal glial cells (Ruzo et al. 2012)
-Although SGSH expression did not lead to an increase in sulfamidase activity in the brain, a (significant) reduction (>30%) in vacuolization has been observed even in 7 month old mice. In contrast Ruzo et al. reported on 2- to 3-fold increases of sulfamidase activity in (non-modified) SGSH expressing animals leading to a significant reduction of GAG storage in the brain. Please comment.
-The statement of the authors, that the brain delivery of ApoB-modified sulfamidase leads to a COMPLETE rescue of brain pathology would require at least measurement of GAG content in the brain

Minor points
-The lack of page numbering is aggravating for comments
-The MPS IIIA mouse resembles only partially the human condition because the Sgsh mutation resulted in residual activity which is not representative for 90% of MPS IIIA patients
-Introduction/Discussion: the authors should included the study by Blanz et al who reported on correction of the brain phenotype in alpha-mannosidosis mice by high-dose replacement with the missing enzyme
-Introduction: IGFI or IGF II?
-General use of small letters: Iduronate-2-sulfatase, Low Density Lipoprotein, ...
-substitute "normal" by "wild-type" mice
-Details on conditioning the medium should be given (composition of the medium; fold concentration)
-The magnification of Fig. 1D has to be increased
-Fig. 1F: I cannot follow the conclusion that IDSsp SGSH and IDSsp (S)GSH-ApoB reach the lysosomal compartment: the merge images show only a low co-localization
- Fig2B is not meaningful: use other colors and higher magnification. Please comment on the effects in hepatocytes/Kupffer cells (incomparison with data of Ruzo et al, 2012)
-Fig 2: explain/discuss the discrepancy between sulfamidase activity in the liver of SGSH-or modified SGSH-MPSIIIA and wild-type GFP mice (2A: 10-fold differences) and the respective serum activities which show only 25% of wild-type or 2-fold higher values, respectively
-Fig 3B: this Figure could be more convincing showing b/w images. Explain and define the abbreviations: ext or deep cortex)
Please comment on discrepancies between data of Ruzo et al showing differences in sulfamidase activity in the liver of treated male and female mice which were not found in the present study.
-Quantify the LCIII blot
-Reference details are incomplete and variable, and the style is not in accordance with the Journal style

Suppl. Material:
Spacing: ApolipoproteinB, IDS sp vs IDSsp
Non-defined/unusual abbreviations: WB, OCT, 10', EM, tol-blue
The usage of the antiFlag M2 antibody has never been described in the text

Referee #2 (General Remarks):

This is a very important paper demonstrating two improvements in enhancing secretion of sulfamidase, using the IDUS signal sequence, and enhancing delivery to brain by targeting the LDLR with a c-terminal tag on the chimeric enzyme. The experiments are well-designed, beautifully illustrated, and the data are significant, well supported, and convincing. The paper should be highly influential in the field and appeal to a broad audience. It is generally well written but a few improvements are suggested.

1) Multiple times the authors use the word uptaken which should be replaced in each instance by

(taken up).

2) Was there a reason for retro orbital injections versus tail vein injections? Could some of the variable response mentioned in the Discussion reflect variability in the efficacy of delivery of the entire injected dose into the retro orbital vein? Good a variable amount be lost by extravasation?

3) In the paragraph under the heading: Apo B-BB modification allows......., Line 6, suggest replacing "instead" with "however" or with "by contrast".

4) In the discussion, the phrase "brain transduction" is used multiple times, which seems inappropriate. I suggest replacing it with "brain delivery" in each instance.

5) On page 2 of discussion, change "which lead" to "which led".

6) On page 3 of the discussion, I suggest deletion of "key" from "additional key factors". Simply saying additional factors is sufficient.

7) Could one of those factors contributing to variability be variable development of antibodies to the gene product? Did the authors measure the immune response to modified enzyme?

8) The last part of the discussion is clumsy and unclear. Suggest breaking it up into two sentences "The data presented should influence developing of future clinical approaches based on AAV2/8-mediated delivery of the modified sulfamidse to MPS IIIA patients. In addition, they raise the possibility of complementing existing ERT protocols for MPS IIIA and other LSDs with enzymes carrying specific brain targeting peptides."

Referee #3 (Comments on Novelty/Model System):

This study is technically sound and is very novel. The experiments have been well thought through. Given the pitfalls associated with gene-therapy based approaches and the relatively slow moving regulatory processes, the medical impact of this study will not be forthcoming immediately. That said, it offers a clever approach to a difficult medical condition and does represent significant progress in the field.

Referee #3 (General Remarks):

This paper focuses on the generation and validation of a gene therapy based approach to treating the neurodegenerative condition Mucopolysaccharidoses type IIA (MPS-IIIA). The authors describe the synthesis of a chimeric sulfamidase gene that has been modified to enhance secretion from the liver, but also to enhance delivery across the blood-brain barrier (BBB). The gene was then packaged into the genome of an adeno-associated virus (AAV) vector, serotype 8 and subsequently injected systemically in a spontaneously occurring mouse model of MPS-IIIA. The principle of the in vivo aspect of the study was that the liver could be used as a "reservoir" for the generation and production of their modified sulfamidase. Given the fact the authors had modified the enzyme to be delivered across the BBB, they go on to show therapeutic efficacy of their system by improving brain pathology and modulating the phenotype of their animal model. The authors propose that their system provides a "feasible strategy to develop minimally invasive therapies for the treatment of brain pathology in MPS-IIIA and other neurodegenerative lysosomal storage diseases (LSDs)".

This is a very interesting paper and the results are convincing and experimental design is well thought through.

Minor Comments
A section in the introduction or discussion describing the choice of AAV serotype....why did the authors choose AAV8? Also, a description of the other delivery mechanisms available would clarify to the reader the choice of vector.
A description of why the authors chose the LDLR receptor at the BBB as opposed to the transferrin receptor to facilitate delivery of their sulfamidase would also help the reader to have a broader "feel"

for the area. For example, a recent study in Science Translational Medicine showed enhanced delivery of antibodies across the BBB when conjugated to the transferrin receptor, with the hypothesis that the interaction between it and its ligand is "weaker" and so activity of the antibody when it reaches the brain is not compromised by too strong an interaction with the receptor (Atwal, J. K. et al. Sci. Trans. Med. 3, 84ra43 (2011) and Yu, Y. J. et al. Sci. Trans. Med. 3, 84ra44 (2011).

Major Comments
One concern related to modifying the sulfamidase to increase its secretion from the liver is the risk of producing too much enzyme. In this regard, what are the "off-target" effects of the enzyme when it is produced systemically, i.e., have the authors looked at pathology of the kidneys, lung, heart etc for any overt signs of toxicity. What about the production of antibodies directed against the sulfamidase...have the authors looked at this?

I don't really understand the controls used in Figure 2B and the labelling needs to be much clearer. Why use AAV2/8-TBG-GFP for controls? Are these empty vectors? The images are very poor quality in Figure 2B and really don't show any staining that's convincing that SSGH is being expressed to a high level. The authors need to include much higher magnification and it would be helpful if they could include some analyses, semi-quantitative or otherwise, regarding the % transduction of the liver.

Again, Figure 3B, the imaging is of poor quality and needs to be much higher magnification. Tissue processing looks to be aberrant as there are numerous holes in the tissue and suggests lack of cryoprotecting or insufficient fixing protocols. That said, there is evident myc-immunoreactivity in some panels. Higher magnification as well as a discussion on the localisation of the staining would be helpful. If the authors had endothelial cell specific antibodies (GLUT-1, CD-31), it would be nice to have some high magnification images of the exact localisation of the myc-reactivity in relation to the microenvironment of the BBB. Also, a comment on the endogenous levels of myc expression in the brain would be good.

In Figure 5B, could the authors analyse frozen section for other signs of "inflammation". Would it be possible to stain for activated microglia using IBA-1 antibodies? Again, higher magnification would be clearer. Also, a double immunostaining for c-myc and GFAP/IBA-1 would be excellent validation that these two events (sulfamidase delivery across the BBB and GFAP decreases) are occurring simultaneously.

Addition to the discussion on the translational potential of this approach would help. The authors should add a section on the current clinical deployment of AAV8...has it been used before? How many injections will a patient need and how often? What about recombinant sulfamidase with the BBB-crossing properties...would this expedite regulation for human therapy as it would by-pass the "gene-therapy" aspect.

1st Revision - authors' response 31 January 2013

Response to reviewers' comments to the manuscript entitled "A highly secreted sulfamidase engineered to cross the blood-brain barrier corrects the CNS pathology of mice with mucopolysaccharidoses type IIIA" by Sorrentino et al.

COMMENTS TO THE REVIEWERS:

-The main figures of the text are 7.
-The previous version of figure 1 was split into two figures (new figures 1 and 2). We added a new paragraph in the text, describing the new figure 2.
-All remaining figures were numbered accordingly:
New figure 3 corresponds to the previous figure 2
New figure 4 corresponds to the previous figure 3
New figure 5 corresponds to the previous figure 4

New figure 6 corresponds to the previous figure 5
New figure 7 corresponds to the previous figure 6
-We added a table (table1)
-We added #2 supplementary figures

POINT BY POINT RESPONSE

Referee #1 (General Remarks):

This is a very interesting and exciting manuscript in which Fraldi and colleagues described for the first time an efficient and minimal invasive gene therapeutic approach to target sulfamidase to the brain of sulfamidase-defective MPSIIIA mice. They generated a modified AAV 2/8 that allow the transduction of the liver and the expression of a sulfamidase construct exhibiting an increased secretion capability and a C-terminal ApoB domain for LDLR-mediated transcytosis of the BBB. The study is a logic continuation of the previous report on early intraventricular injection of SGSH in MPSIIIA mice resulting, however, in a higher long-lasting efficacy, which might be applicable to other lysosomal storage disorders affecting the brain.

The presentation and discussion of the data, however, require substantial improvement and more precise and informative data sets before publication. In general, the authors should describe all parts of the different Figures in more detail and not only those, which are confirmatory.

Following the reviewer's suggestions we have now carefully edited the manuscript. We added new references and provided more precise data sets. We added more information in all the figures to describe more clearly the experiments performed.

Major concerns and specific comments:

-Fig 1B: indicate the molecular masses and describe the expected masses of sulfamidase constructs; explain differences between cellular and secreted forms. It is unclear whether or not the cellular forms are processed in the lysosomes. A single blot only will allow the comparison of the different forms but is still misleading to determine the secretion rate. The advantage of the IDSsp on secretion is not obvious and requires pulse-chase experiments.

Following the suggestions of referee 1 we have performed pulse&chase experiments on cells transfected with different SGSH constructs (see Methods section for details) to analyse better the maturation and secretion of the modified sulfamidase. These experiments are now showed in the new figure 1B and replaced the single blot showed in the previous version of the figure 1B.

As illustrated in the figure 1B, the precursor forms of WT and partially modified SGSH (IDSspSGSH) could be detected in the cell pellet as proteins with a molecular mass of approximately 62 KDa. After 12 and 24 hours of chase these enzymes were processed in their corresponding mature forms (≈ 56 kDa). The addition of the ApoB binding domain (BD) resulted in a slight increase in the size (≈ 3 KDa shift) of the band corresponding to the IDSspSGSH-ApoB chimeric enzyme (new Figure 1B). An additional band with a molecular mass greater than that we expected by the addition of the ApoB-BD was detected at ≈ 70 kDa (indicated by the asterisk the new Figure 1B). This band could correspond to a precursor form of the ApoB-BD-modified SGSH with a different glycosylation pattern (this explaining the observed further increase in the size). However, this conclusion should be confirmed by more specific analyses. From 12 hours of chase the precursor forms of all SGSH enzymes started to appear in the medium (new figure 1B). Notably, the ApoB-BD-modified SGSH was mainly secreted as the 70 kDa protein (new Figure 1B)

consistently with the hypothesis that this protein corresponds to a precursor form of the fully modified SGSH.

Moreover, the ratio of secreted proteins (in the medium) to total protein (cells + medium) at each chase time demonstrated that the addition of the IDS sp led to an increase in the secretion rate of the modified sulfamidase enzyme compared to wild-type sulfamidase (new Figure 1C).

-Results: The authors should provide specific sulfamidase activity values for the pellet and medium rather than relative activity (provide fold-increases in activity, distribution and uptake for all conditions

We followed the reviewer's suggestion and converted the relative activity in nanomole of enzyme/17 hours of incubation/milligrams of protein (pellet activity) or nanomole of enzyme/17 hours of incubation/millilitres (medium activity).

-The description of the experimental setup shown in Fig. 1E is rather cryptic. Controls in the presence of M6P or LDL are missing to show the specificity of uptake and the dominance of individual targeting signals in SGSH-ApoB.

How much sulfamidase (mU) was offered? According to the Figure legend and the text in the Results part the authors offered equal amounts of conditioned media to Hepa cells which could explain the increase in uptake of modified SGSH, or why should the uptake of IDSsp SGSH (after loss of the signal peptide in the ER) be significantly (3-fold) higher than wild-type SGSH?

We agree with the reviewer that the experimental setup showed in the previous figure 1E was not clear and do not fully support the conclusions we made.

Following the reviewer's suggestions we performed the following uptake experiments:

1) Recipient cells (MPS-IIIA MEFs) were incubated with conditioned medium (derived from Hepa transfected cells) containing similar specific enzymatic activities (20 nmol/17/ml) and not with equal amount of conditioned medium. This led us to determine the efficiency of the uptake independently from the amount of the enzyme present in the conditioned medium.

2) We measured the uptake in the presence of M6P to determine the contribution of M6P receptor-mediated endocytosis in the uptake of the ApoB-BD containing chimeric SGSH.

The results of these experiments are showed in the table 1. We have also added further explanations and experimental details in both the main text and Methods section.

-The authors should provide data on the uptake of sulfamidase in other non-neuronal cells (Fig. 3C) because the MPS pathology has been reported to be more evident in perineuronal glial cells (Ruzo et al. 2012).

The previous figure 3C (now new figure 4C) indirectly showed that the ApoB-BD-containing chimeric SGSH is taken up by non-neuronal cells (NeuN-negative staining). However, to directly demonstrate the specificity of the perineuronal glia cells uptake we followed the reviewer's suggestion and performed an immunofluorescence staining on brain sections derived from IDSspSGSH-ApoB injected MPS-IIIA mice using an anti GFAP antibody, that is specific for astroglia. This staining is showed in the new figure 4C and demonstrated that the ApoB-BD-modified SGSH was taken up by astroglial cells.

-Although SGSH expression did not lead to an increase in sulfamidase activity in the brain, a (significant) reduction (>30%) in vacuolization has been observed even in 7 month old mice. In contrast Ruzo et al. reported on 2- to 3-fold increases of sulfamidase activity in (non-modified) SGSH expressing animals leading to a significant reduction of GAG storage in the brain. Please

comment.

The extent of vacuolization (% of cells with pathological vacuoles) in the MPS IIIA mice injected with either wild-type or partially modified sulfamidase (IDSspSGSH) decreased only slightly at 7 months after injection compared to control GFP-injected MPS-IIIA mice (from 45% observed in control GFP-injected MPS-IIIA mice to approximately 32% in both wild-type and partially modified sulfamidase-injected MPS-IIIA). This decrease is not significant (the statistics are now showed in the new figure 5A). Conversely, the vacuolization strongly decreased in the MPS-IIIA mice injected with the fully modified sulfamidase compared to control GFP-injected MPS-IIIA mice (from 45% to 6-7%). We concluded that in agreement with the absence of SGSH enzymatic recovery, any significant reduction of pathological vacuolization occurred in the brain of MPS-IIIA injected with either wild-type or partially modified sulfamidase. Furthermore, as highlighted by the reviewer, Ruzo et al reported on 2- to 3-fold increases of sulfamidase activity in (non-modified) SGSH expressing animals leading to a significant reduction of GAG storage in the brain. In fact, we discussed this discrepancy in the manuscript (see Discussion section).

-The statement of the authors, that the brain delivery of ApoB-modified sulfamidase leads to a COMPLETE rescue of brain pathology would require at least measurement of GAG content in the brain.

As suggested by the reviewer, we measured the GAG content in the brain homogenates of the mice injected with fully modified sulfamidase to support our conclusions. Consistent with the recovery of sulfamidase activity, the MPS-IIIA mice injected with fully modified sulfamidase showed a clearance of accumulated GAGs that were reduced to levels similar to those observed in control normal mice. The results were now showed in the supplementary figure 3.

Minor points

-The lack of page numbering is aggravating for comments

We have now added page numbering.

-The MPS IIIA mouse resembles only partially the human condition because the Sgsh mutation resulted in residual activity, which is not representative for 90% of MPS IIIA patients.

We completely agree with the reviewer. Indeed, when we mentioned the similarities between the human condition and MPS IIIA mouse model we referred to the pathophysiology and disease progression and not to the residual sulfamidase activity.

-Introduction/Discussion: the authors should include the study by Blanz et al who reported on correction of the brain phenotype in alpha-mannosidosis mice by high-dose replacement with the missing enzyme.

As suggested by the reviewer, we now mentioned this study in the discussion and added the correspondent reference.

-Introduction: IGFI or IGF II?

IGF II.

-General use of small letters: Iduronate-2-sulfatase, Low Density Lipoprotein, ...

-substitute "normal" by "wild-type" mice

We thank the reviewer for his/her suggestions and edited the manuscript accordingly.

As now better specified in both the main text and Methods section we did not use wild-type (sgsh+/+) mice but heterozygous (sgsh+/-) mice. These mice are phenotypically normal (Bhattacharyya et al, 2001; Bhaumik et al, 1999; Fraldi et al, 2007). Therefore the term "normal mice" is used to refer to the mouse phenotype and not to the mouse genotype.

-Details on conditioning the medium should be given (composition of the medium; fold concentration).

These details were added in the Methods section.

-The magnification of Fig. 1D has to be increased

We modified the figure accordingly.

-Fig. 1F: I cannot follow the conclusion that IDSspSGSH and IDSspSGSH-ApoB reach the lysosomal compartment: the merge images show only a low co-localization

The poor quality/resolution of the pictures in the previous figure 1F we used in the first submission do not allow appreciating the co-localization between the modified SGSH and the lysosomal compartment. We have now replaced these pictures with new high-resolution images and also added magnification images showing the presence of sulfamidase into LAMP1-positive structure (new figure 2)

-Fig2B is not meaningful: use other colours and higher magnification. Please comment on the effects in hepatocytes/Kupffer cells (in comparison with data of Ruzo et al, 2012).

We realized that the colours of the images in the previous version of figure 2B were quite confusing due to the low resolution of the figure images sent in this first submitted version of the manuscript. According to the reviewers suggestion we used images with higher resolution and new colours to better visualize sulfamidase staining. Moreover magnification images were added in the new figure 3A (previous figure 2B). .

-Fig 2: explain/discuss the discrepancy between sulfamidase activities in the liver of SGSH-or modified SGSH-MPSIIIA and wild-type GFP mice (2A: 10-fold differences) and the respective serum activities, which show only 25% of wild-type or 2-fold higher values, respectively.

The reviewer is right. We explain this discrepancy by the assumption that the exogenous sulfamidase secreted from the liver of the MPS-IIIA mice injected with different SGSH constructs is partially cleared due the its uptake by other organs (which are deficient for functional and active sulfamidase) and by the liver itself. This clearance is not effective in control wild-type mice in which all the organs, included the liver, stably express the endogenous sulfamidase enzyme. We now discuss this in the text (Discussion section).

-Fig 3B: this Figure could be more convincing showing b/w images. Explain and define the abbreviations: ext or deep cortex).

Following the reviewer's suggestion we used black&white images in the new figure 4B (previous figure 3B). The abbreviation "ext cortex" is referred to the superficial layers (molecular and granular layers) while "deep cortex" is referred to the deep layers containing pyramidal cells.

Please comment on discrepancies between data of Ruzo et al showing differences in sulfamidase activity in the liver of treated male and female mice, which were not found in the present study.

As mentioned by the reviewer we did not observe significant differences in the levels of liver sulfamidase activity between female and male MPS-IIIA mice. Notably, other labs have reported on the absence of differences in the expression of AAV2/8-mediated delivered lysosomal enzymes between male and female MPS mice (e. g. Cardone et al 2007)

It still remains to determine which are the underlying mechanisms generating the differences observed by Ruzo et al. Surprisingly, Ruzo et al did not discuss this point in their manuscript.

-Quantify the LCIII blot

We quantified the LC3-II blot.

-Reference details are incomplete and variable, and the style is not in accordance with the Journal style

We edited reference details in accordance with the Journal style.

Suppl. Material:

Spacing: ApolipoproteinB, IDS sp vs IDSsp

Non-defined/unusual abbreviations: WB, OCT, 10', EM, tol-blue

The usage of the antiFlag M2 antibody has never been described in the text

We edited the manuscript according the reviewer's suggestions. Moreover, we specified in the main text that all the SGSH constructs generated contained a myc-flag tag (Fig. 1A). We now described the use of anti-flag antibody in the main text (first paragraph in the Results section).

Referee #2 (General Remarks):

This is a very important paper demonstrating two improvements in enhancing secretion of sulfamidase, using the IDUS signal sequence, and enhancing delivery to brain by targeting the LDLR with a c-terminal tag on the chimeric enzyme. The experiments are well designed, beautifully illustrated, and the data are significant, well supported, and convincing. The paper should be highly influential in the field and appeal to a broad audience. It is generally well written but a few improvements are suggested.

1) Multiple times the authors use the word uptaken which should be replaced in each instance by

(taken up).

We replaced the word "uptaken" with "taken up".

2) Was there a reason for retro orbital injections versus tail vein injections? Could some of the variable response mentioned in the Discussion reflect variability in the efficacy of delivery of the entire injected dose into the retro orbital vein? Good a variable amount be lost by extravasation?

We used the retro orbital injection since in a comparative study performed in MPS IIIA mice we observed that this type of injection is more reliable than the tail vein injection.

3) In the paragraph under the heading: Apo B-BB modification allows......., Line 6, suggest replacing "instead" with "however" or with "by contrast".

4) In the discussion, the phrase "brain transduction" is used multiple times, which seems inappropriate. I suggest replacing it with "brain delivery" in each instance.

5) On page 2 of discussion, change "which lead" to "which led".

6) On page 3 of the discussion, I suggest deletion of "key" from "additional key factors". Simply saying additional factors is sufficient

We revised the manuscript following all the reviewer's suggestions (points 3, 4, 5 and 6) by modifying the test accordingly.

7) Could one of those factors contributing to variability be variable development of antibodies to the gene product? Did the authors measure the immune response to modified enzyme?

We agree with the reviewer that evaluating the presence of an immune response against the modified enzyme could be helpful to determine the factors that contribute to the variability we detected in brain transduction. However, we observed very high and sustained levels of modified sulfamidase in both liver and serum of injected MPS-IIIA mice up to 7 months post-injection. This indirectly indicates that, a potential immune response against the chimeric sulfamidase would have been very low or not effective.

8) The last part of the discussion is clumsy and unclear. Suggest breaking it up into two sentences "The data presented should influence developing of future clinical approaches based on AAV2/8-mediated delivery of the modified sulfamidse to MPS IIIA patients. In addition, they raise the possibility of complementing existing ERT protocols for MPS IIIA and other LSDs with enzymes carrying specific brain targeting peptides."

We agree with the reviewer and modified the last part of the discussion accordingly.

Referee #3 (General Remarks):

This paper focuses on the generation and validation of a gene therapy based approach to treating the neurodegenerative condition Mucopolysaccharidoses type IIA (MPS-IIIA). The authors describe the synthesis of a chimeric sulfamidase gene that has been modified to enhance secretion from the liver, but also to enhance delivery across the blood-brain barrier (BBB). The gene was then packaged into the genome of an adeno-associated virus (AAV) vector, serotype 8 and subsequently injected systemically in a spontaneously occurring mouse model of MPS-IIIA. The principle of the in vivo

aspect of the study was that the liver could be used as a "reservoir" for the generation and production of their modified sulfamidase. Given the fact the authors had modified the enzyme to be delivered across the BBB, they go on to show therapeutic efficacy of their system by improving brain pathology and modulating the phenotype of their animal model. The authors propose that their system provides a "feasible strategy to develop minimally invasive therapies for the treatment of brain pathology in MPS-IIIA and other neurodegenerative lysosomal storage diseases (LSDs)".

This is a very interesting paper and the results are convincing and experimental design is well thought through.

Minor Comments

A section in the introduction or discussion describing the choice of AAV serotype….why did the authors choose AAV8? Also, a description of the other delivery mechanisms available would clarify to the reader the choice of vector.

Following the reviewer's suggestion we have now added a section in the discussion that describes the choice of the vector (AAV serotype 8) in the context of other delivery vehicles.

A description of why the authors chose the LDLR receptor at the BBB as opposed to the transferrin receptor to facilitate delivery of their sulfamidase would also help the reader to have a broader "feel" for the area. For example, a recent study in Science Translational Medicine showed enhanced delivery of antibodies across the BBB when conjugated to the transferrin receptor, with the hypothesis that the interaction between it and its ligand is "weaker" and so activity of the antibody when it reaches the brain is not compromised by too strong an interaction with the receptor (Atwal, J. K. et al. Sci. Trans. Med. 3, 84ra43 (2011) and Yu, Y. J. et al. Sci. Trans. Med. 3, 84ra44 (2011).

In the introduction we mentioned the different BBB receptors that may be potentially useful to target proteins to the brain via BBB trancytosis. We believe that the potential therapeutic efficacy of these other systems should be explored in further studies. Here, we have chosen the LDLR as delivery system on the basis of previous studies that demonstrated the efficacy of this system in allowing BBB crossing of a lisosomal hydrolytic enzyme (Spencer & Verma, 2007; Spencer et al 2011). Following the reviewer's suggestion we also mentioned in the introduction the study on transferrin receptor-mediated delivery of antibodies across the BBB and added the correspondent references (Atwal, J. K. et al. Sci. Trans. Med. 3, 2011; and Yu, Y. J. et al. Sci. Trans. Med. 3, 2011).

Major Comments

One concern related to modifying the sulfamidase to increase its secretion from the liver is the risk of producing too much enzyme. In this regard, what are the "off-target" effects of the enzyme when it is produced systemically, i.e., have the authors looked at pathology of the kidneys, lung, heart etc for any overt signs of toxicity. What about the production of antibodies directed against the sulfamidase...have the authors looked at this?

We share the concerns raised by the reviewer. Regarding this issue, there are several studies that report the use of blood system overloading to increase the amount of enzyme available to target the brain. We did not observe the presence of obvious signs of pathophysiology in different "off-targets" organs such as kidney, spleen, heart and lung. However, as commented by the reviewer we cannot exclude the occurrence of some toxic effects especially at long term. The evaluation of these effects should be addressed in future studies. These studies might constitute an important set of data needed to evaluate the potential clinical translation of the approach developed in the present work. This point is now discussed in the text (see Discussion section).

Regarding the immune response. We did not look at the production of antibodies against the modified sulfamidase enzymes. Again, we recognize the relevance of this aspect in generating further preclinical data propaedeutic for the clinical translation of the approach. However, we

observed very high and sustained levels of modified sulfamidase in both liver and serum of injected MPS-IIIA mice up to 7 months post-injection. This indirectly indicates that, a potential immune response against the chimeric sulfamidase was very low or not effective.

I don't really understand the controls used in Figure 2B and the labelling needs to be much clearer. Why use AAV2/8-TBG-GFP for controls? Are these empty vectors? The images are very poor quality in Figure 2B and really don't show any staining that's convincing that SSGH is being expressed to a high level. The authors need to include much higher magnification and it would be helpful if they could include some analyses, semi-quantitative or otherwise, regarding the % transduction of the liver.

We used empty plasmids as controls in the in vitro studies (old Figure 1 split in new figures 1 and 2). In the in vivo studies wild-type and MPS-IIIA mice were injected with AAV2/8-TBG-GFP as controls.

We realized that the colours of the images in the previous version of figure 2B were quite confusing due to the low resolution of the figure images sent in this first submitted version of the manuscript. According to the reviewers suggestion we used images with higher resolution and new colours to better visualize sulfamidase staining. Moreover magnification images were added in the new figure 3A (previous figure 2B). .

Again, Figure 3B, the imaging is of poor quality and needs to be much higher magnification. Tissue processing looks to be aberrant as there are numerous holes in the tissue and suggests lack of cryoprotecting or insufficient fixing protocols. That said, there is evident myc-immunoreactivity in some panels. Higher magnification as well as a discussion on the localization of the staining would be helpful. If the authors had endothelial cell specific antibodies (GLUT-1, CD-31), it would be nice to have some high magnification images of the exact localization of the myc-reactivity in relation to the microenvironment of the BBB. Also, a comment on the endogenous levels of myc expression in the brain would be good.

We used higher resolution images in the new figure 4B (previous figure 3B). Moreover, we used black&white images to better highlight the immunoreactivity of the myc-tagged proteins. As suggested by the reviewer we performed an immunofluorescence against GLUT1 on the brain sections of mice injected with AAV2/8-TBG-IDSspSGSH-ApoB to study the localization of the modified enzyme in the context of BBB endothelial cells. The results of these experiments are now showed in the new Fig. 4 D. As mentioned by the reviewer the expression of the endogenous myc could raise a background signal. We reduced/masked this background signal by using ammonium chloride 50 mM after fixation (see Methods section).

In Figure 5B, could the authors analyse frozen section for other signs of "inflammation". Would it be possible to stain for activated microglia using IBA-1 antibodies? Again, higher magnification would be clearer. Also, a double immunostaining for c-myc and GFAP/IBA-1 would be excellent validation that these two events (sulfamidase delivery across the BBB and GFAP decreases) are occurring simultaneously.

As suggested by the reviewer we performed an immunofluorescence using anti-IBA-1 antibodies to evaluate the reduction of inflammation signs (activated microglia) in the brain of MPS-IIIA mice upon the injection of the fully modified sulfamidase. These results are now showed in the supplementary figure 4.

Addition to the discussion on the translational potential of this approach would help. The authors should add a section on the current clinical deployment of AAV8...has it been used before? How many injections will a patient need and how often? What about recombinant sulfamidase with the BBB-crossing properties...would this expedite regulation for human therapy as it would by-pass the "gene-therapy" aspect.

As suggested by the reviewer we now added a part in the discussion that describes the current clinical deployment of AAV2/8 in the context of the choice of AAV2/8 respect to other viral-mediated liver-directed systems. Moreover, we expanded the part of the discussion that describes the translation potential of the therapeutic approach developed in this study.

2nd Editorial Decision 19 February 2013

Thank you for the submission of your revised manuscript to EMBO Molecular Medicine. We have now received the enclosed reports from the Reviewers that were asked to re-assess it. As you will see the reviewers are now globally supportive and I am pleased to inform you that we will be able to accept your manuscript pending the final amendments:

1) Reviewer 1 points to a few remaining issues (textual) that you need to properly deal with in the final version.

2) I have noticed that while images are generally of good quality, the text in many figures is rather blocky/blurry and does not hold well to magnification. Please provide higher resolution versions, and check to make sure that text/line-art remains clear even when zooming in. You may find that saving the images as EPS or PDF will better preserve the text and line-art resolution. If this does not help, you may need to remake the figures in a quality vector graphics program like Illustrator or the free opensource, alternative Inkscape. Furthermore, the track plots in Fig. 7 (A and C) are also quite blurry. Please try improving them.

3) I would like to suggest the following alternative title for your paper to increase impact: "A highly secreted sulfamidase engineered to cross the blood-brain barrier corrects brain lesions in mice with mucopolysaccharidosis type IIIA". Would this be acceptable? Would you like to propose a shorter alternative?

4) Please verify if you can supply the information described at item 6) below.

Please submit your revised manuscript within two weeks. Needless to say, the sooner we receive it the sooner I will be able to formally accept your manuscript.

I look forward to reading a new revised version of your manuscript as soon as possible.

***** Reviewer's comments *****

Referee #1 (General Remarks):

The revised manuscript answered all open questions of the first version in a sufficient manner. The authors provided new experimental data which supported their conclusions and improved the quality of the manuscript. There are, however, some minor imprecise terms and phrase which should be clarified or substituted, without impairing my recommendation for acceptance of the manuscript for publication:

1. There is an objection in the Introduction p4, line 10, describing the "reduced expression of M6P receptors on the BBB" (they mean the expression in cells forming the BBB) and p4, lines 15-18: .. by receptors that are highly and age-independently enriched on the BBB....include the LDLR, TfR and the insulin-like growth factor receptor II.
The M6P receptor and the insulin-like growth factor receptor II, however, are identical proteins!
2. pp8/throughout the manuscript (incl. legend to Fig4) the authors write:
... MPSIIIA mice injected with fully/partially modified (wildtype) sulfamidase..they mean ...mice expressing (in the liver) the fully/partially......
3. p16 subheading: Immunofluorescence microscopy
4. Table 1 requires the unit definition of activity in recipient MEFs

Referee #2 (Comments on Novelty/Model System):

This manuscript is greatly improved, has addressed all concerns I had and I feel it addresses concerns of other reviewers. Favor expedited publication because of high impact and importance to the field. Will be widely read and influential.

Referee #2 (General Remarks):

Greatly improved and addresses all relevant concerns. Bravo!

Referee #3 (Comments on Novelty/Model System):

The authors have addressed all of the comments from my previous review of the manuscript

Referee #3 (General Remarks):

The authors have addressed all of my concerns and have done a good job in providing extra experimental data for both mine and the other reviewers comments. I look forward to seeing their paper in press.

2nd Revision - authors' response 20 February 2013

Please find enclosed our revised manuscript entitled "A highly secreted sulfamidase engineered to cross the blood-brain barrier corrects brain lesions of mice with mucopolysaccharidoses type IIIA", by Sorrentino et al.

We modified the text according to the suggestions requested by reviewer 1 and we modified the text and the figures taking into account the *EMBO Molecular Medicine* instructions.

Following the editorial suggestions we also modified the title from "A highly secreted sulfamidase engineered to cross the blood-brain barrier corrects the CNS pathology of mice with mucopolysaccharidoses type IIIA" to "A highly secreted sulfamidase engineered to cross the blood-brain barrier corrects brain lesions of mice with mucopolysaccharidoses type IIIA". Moreover, we also provided higher resolution images saved as pdf files to preserve the text and line-art resolution.

We hope that this new version of the manuscript is now appropriate for its ultimate publication in *EMBO Molecular Medicine*
