# Supplementary material for: A highly secreted sulphamidase engineered to cross the blood-brain barrier corrects brain lesions of mice with mucopolysaccharidoses type IIIA
Source: EMBO Mol Med. 2013 Apr 9;5(5):675–90. doi: 10.1002/emmm.201202083 (PMC3662312; doi:10.1002/emmm.201202083)
Supplement: Supplementary file 2 [file emmm0005-0675-sd2.pdf]

SUPPORTING INFORMATION

Manuscript entitled "A highly secreted sulfamidase engineered to cross the blood-brain barrier corrects the CNS pathology of mice with mucopolysaccharidoses type IIIA" by Sorrentino et al.

TABLE OF CONTENT:

-Supplementary Methods

-Supplementary Figures

-Legends to Supplementary Figures

-Supplementary Methods

Construction of chimeric sulfamidase constructs and AAV vectors

The hSGSH signal peptide (sp) was replaced by the IDS sp by ligating two fragments: A sequence from human SGSH cDNA (hSGSH) and the IDS signal peptide sequence (IDS sp). The hSGSH was amplified from an expressing plasmid beginning at the 3' terminus of hSGSH signal peptide sequence and extending to a unique XbaI site. The IDS sp was synthesized by annealing of specific oligonucleotide sequences (forward and reverse) containing the IDS sp sequence [Homo sapiens iduronate 2-sulfatase (IDS) cDNA: position 218-292 (75 bp)]. The two oligonucleotide sequences have 5' NotI site and 3' blunt end site:

Forward, 5'- GGC CGC ATG CCC CCG CCC CGC ACC GGC CGC GGC CTG CTG TGG CTG GGC CTG GTG CTG AGC AGC GTG TGC GTG GCC CTG GGC -3'. Reverse, 5'- GCC CAG GGC CAC GCA CAC GCT GCT CAG CAC CAG GCC CAG CCA CAG CAG GCC GCG GCC GGT GCG GGG CGG GGG CAT GC-3'

To obtain the fully modified SGSH chimeric construct, the Binding Domain of the human Apolipoprotein B (aminoacids 3371-3409) was cloned into the BglII sites at 5' terminus of 3xFlag tag of IDSspSGSHFlag. The 3xFlag tag was replaced by a MycFlag in all constructs. Therefore, the different expression cassettes containing the unmodified sulfamidase, the partially modified constructs (only IDS sp replacement) and the fully modified construct (with both IDS sp and ApoB-BD) were subcloned in the pAAV2.1-TBG-expression plasmids to generate the correspondent AAV serotype 8 (AAV2/8) viral vectors according protocols established at AAV TIGEM Vector Core.

LC3 analysis

Brain samples were incubated with Lysis buffer (50 mM Tris-HCl pH8, 200 mM NaCl, 1% Triton X100, 1 mM EDTA, 50 mM HEPES) for 1 hour in ice. Protein concentration was determined using the Bio-Rad (Bio-Rad, Hercules, CA, USA) colorimetric assay. Polyclonal anti-LC3 (Novus Biologicals) was used to reveal LC3-II in brain samples.

Tissue collection

Mice were euthanized, blood samples were collected and allowed at room temperature prior to centrifugation at 10.000 g x 10'at 4ºC to obtain the serum. Serum was harvested and stored frozen until use. To collect liver and brain, mice from each experimental group were perfused with phosphate-buffered saline (PBS pH 7,4) to completely clear blood from tissue. Brain and liver was divided in two equal part: an half part of each was frozen in dry ice and the other half was fixed in 4% (w/v) paraformaldehyde in PBS and embedded in OCT matrix (for immune-staining) or in 4% paraformaldehyde, 25% glutaraldehyde in phosphate buffer (for EM).

GAG quantification

Brain samples were lysed in water by Tissue Lyser equipment (Qiagen, Netherlands) and then by 3 cycles of freeze/thaw. The lysetes were then digested with proteinase K and extracts were clarified by centrifugation and filtration. GAG levels in brain extracts were determined using Blyscan sulfated glycosaminoglycan kit (Biocolor, Carrickfergus, UK) with chondroitin 4-sulfate as the standard.

-Supplementary Figure 1

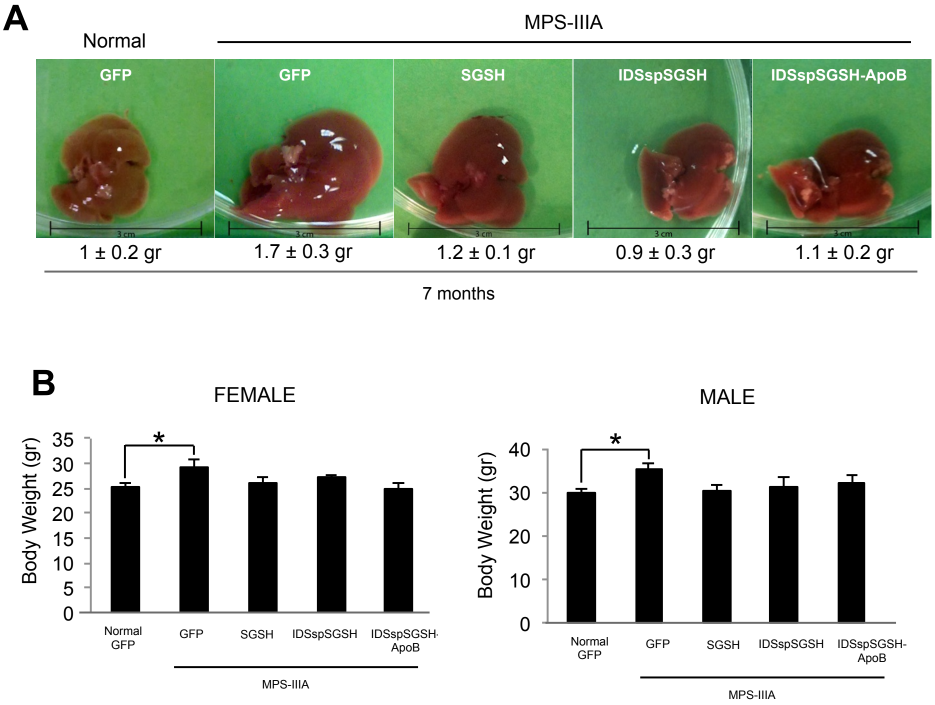

-Supplementary Figure 2

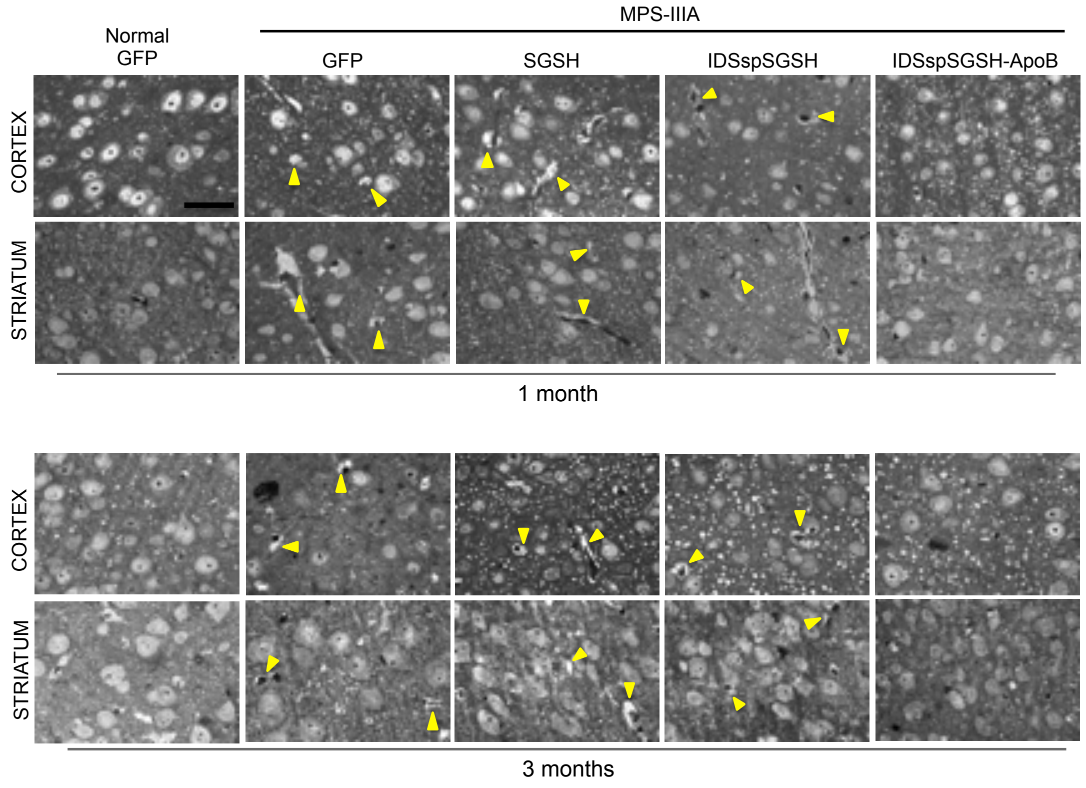

-Supplementary Figure 3

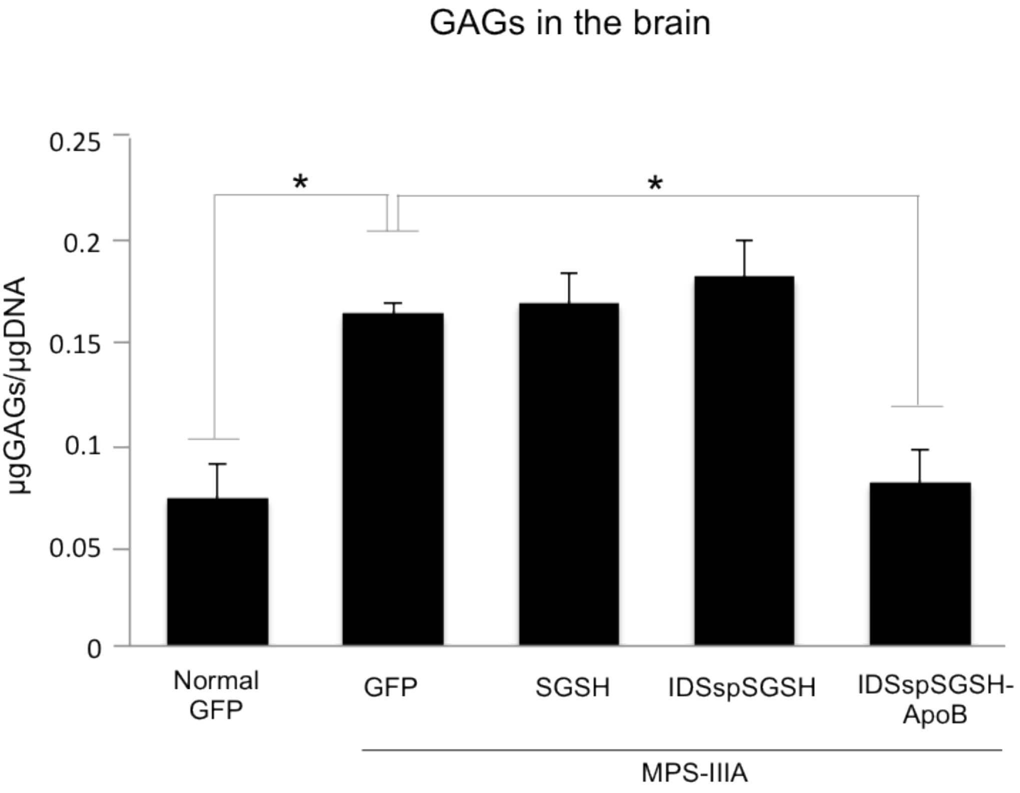

-Supplementary Figure 4

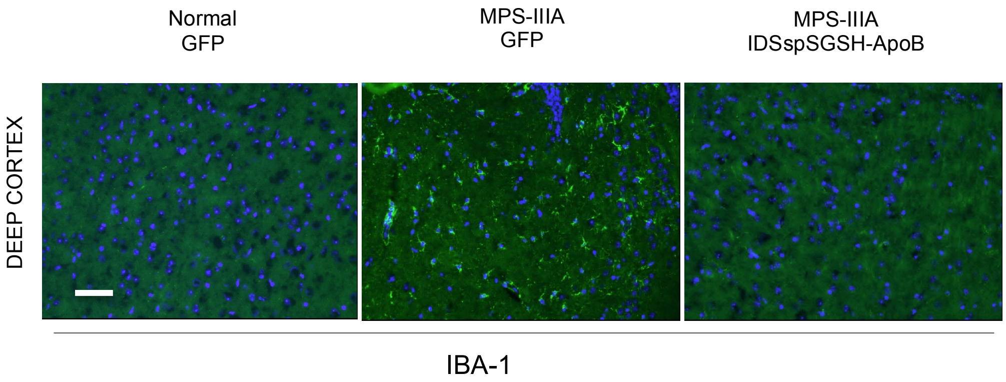

-Legends to Supplementary Figures

Figure S1. Liver mass and body weight upon AAV2/8-TBG-medited delivery of the engineered sulfamidase constructs. (*A*) Size and weight of livers collected from the indicated experimental group of mice at 7 months after the treatment. Weight (gr) ± S.E.M. (*B*) Mean body weight (gr ± S.E.M) measured in female and male mice 7 months after the indicated treatments. Five animals were analysed for each experimental group. * $P < 0.05$ (vs normal-GFP), Bonferroni-Dunnet post-hoc test.

Figure S2. Pathological vacuolization in the brain of treated MPS-IIIA mice. Ultra-thin brain sections from the indicated experimental group of mice at 1 and 3 months after injection were stained with tol-blue to evaluate extent of vacuolization (yellow arrowheads indicated extensive vacuolated cells). Scale bar, 30 µm

Figure S3. GAG content in the brain of treated MPS-IIIA mice. GAGs were quantified in brain samples derived from the indicated experimental group of mice at 7 months after injection. GAGs were measured using a colorimetric assay as indicated in the Methods section and were expressed as µg of GAGs normalized to µg DNA in the sample. Values are means ± SEM (triplicate experiments) * $P < 0.05$ Student's t-test (Normal GFP vs MPS-IIIA SGSH, MPS-IIIA SGSH vs MPS-IIIA IDSspSGSH and MPS-IIIA SGSH vs MPS-IIIA IDSspSGSH-ApoB).

Figure S4. Activated microglia in treated MPS-IIIA mice. Brain cryosections from the indicated experimental group of mice were stained with anti-IBA-1 (green) to evaluate the presence of activated microglia in the brain of treated mice. DAPI (blue) stains nuclei. Scale bar, 30 µm.
